# A multivariate study on the effect of 3D printing parameters on the printability of gluten‐free composite dough

**DOI:** 10.1002/jsfa.14031

**Published:** 2024-11-28

**Authors:** Abhishek Pradhan, Debapam Saha, Punyadarshini Punam Tripathy

**Affiliations:** ^1^ Agricultural and Food Engineering Department, Indian Institute of Technology Kharagpur Kharagpur India

**Keywords:** 3D food printing, gluten‐free dough, rheological analysis, printability assessment, principal component analysis, textural properties

## Abstract

**BACKGROUND:**

The advancement of three‐dimensional (3D) food printing technology is significantly influencing the food processing industry. The present study utilized extrusion 3D printing to create a gluten‐free dough composed of little millet flour (LMF), amaranth seed flour (ASF) and curry leaf flour (CLF). The primary objective was to elucidate the effects of various extrusion‐based 3D printing conditions, including extruder nozzle diameter (*ND*), extrusion rate (*ER*), print speed (*PS*) and layer height (*LH*) on the printability parameters of the dough.

**RESULTS:**

In this study, three formulations of composite dough were prepared (i.e., S1, S2 and S3) by varying LMF, ASF and CLF compositions; among which S1 composite (LMF:ASF:CLF::30:60:10 w/w) was found to have comparable viscoelastic properties with the wheat dough. Central composite design (CCD) was selected with 30 experimental runs and 3D dough samples were printed using S1 composite by varying *ND*, *ER*, *PS* and *LH*. Principal component analysis of the printed samples accounted for 82.75% of variability and classified the samples into three confidence ellipses. From the results of the CCD model, four solution parameters in terms of *ND*:*ER*:*PS*:*LH*::mm: pulse μL^−1^:mm s^−1^:%, namely, *P*_1_ (1.8:115:8:70), *P*_2_ (1.6:115:7.8:65), *P*_3_ (2:100:7.8:55) and *P*_4_ (2:105:6.2:75), with high desirability value (> 0.85) were selected for sample 3D printing and post‐baking analysis. The *P*_1_ sample was found to have least print deformations, highest print fidelity (85.66%), and lowest hardness (55.95 N) and fracturability (39.66 N) values, and hence was selected as optimized product.

**CONCLUSION:**

The present study provides parametric solutions for efficient 3D printing of gluten‐free composite dough, aligning with the increasing dietary preferences and demand of novel functional customized foods. © 2024 The Author(s). *Journal of the Science of Food and Agriculture* published by John Wiley & Sons Ltd on behalf of Society of Chemical Industry.

## INTRODUCTION

Millets and pseudocereals are being increasingly used in food research to develop nutritionally enriched novel food products.^1^, ^2^ They are naturally gluten‐free and can be advantageous for people with celiac disease or gluten sensitivity, offering a healthy substitute for wheat‐based products. Little millet (*Panicum miliare*) is a minor millet, abundant in antioxidants, phytochemicals and minerals, and is regarded as a low glycemic index food because it contains a substantial amount of dietary fiber.^3^ Similarly, amaranth seed (*Amaranthus caudatus* L.) is a pseudocereal, having an exceptional nutritional value, and provides essential vitamins such as vitamin E, thiamine, niacin, riboflavin and folate, along with minerals such as iron, calcium, magnesium, phosphorus, zinc and manganese, as well as high‐quality protein rich in lysine and methionine amino acids.^4^ Also, curry leaf (*Murraya koenigii*) known for its rich bioactive profile can contribute to the overall consistency by acting as a natural binder when used in gluten‐free dough formulation.^5^ As a result, significant research and commercial efforts have increasingly focused on creating innovative value‐added products from gluten‐free components over the years, such as pearl millet‐based sourdough breads,^6^ teff‐soyabean based extrudates^7^ and buckwheat‐proso millet‐based baked product.^8^

Among the various advanced food development practices, the modern 3D printing technology is finding application in the bakery industry because of its capability to fabricate intricate shapes and textures to cater gluten‐free dietary preferences.^9^, ^10^, ^11^, ^12^, ^13^ However, gluten‐free formulations face challenges in 3D printing, resulting in poor dough elasticity, weak structural integrity and difficulties in maintaining shape during the printing process. For example, quinoa flour and buckwheat flour, which are rich in protein and fibre, add nutritional value to printed foods, but they affect the paste's consistency and are more challenging to print because of their dense texture and tendency to form lumps.^14^, ^15^ Furthermore, it is very crucial to track the printability of gluten‐free formulations in terms of structural changes after the printing process.^16^

Printability evaluation includes assessing extrudability, structural stability and dimensional accuracy.^17^ Some of the printability assessment techniques include image analysis through ImageJ^18^ and optical 3D scanning,^19^ or by manual measurements of structural geometry and their further multivariate characterization using multi‐response modelling,^20^ principal component analysis^21^ and correlation analysis.^22^ However, these printability characteristics of the paste are significantly influenced by the printing parameters including nozzle diameter, extrusion rate, nozzle head speed and layer height. The nozzle diameter impacts the printing precision of the extruded object because smaller nozzles provide finer details but increase the risk of clogging, especially with viscous doughs.^23^ Similarly, the nozzle head speed affects the dough extrudability, where faster speed reduces the production time but potentially compromises the structural integrity of the printed layers.^24^ Also, layer height influences the surface smoothness and appearance of the printed product, with thinner layers providing finer detail but simultaneously extending the printing time.^25^ Therefore, optimizing the printing process parameters is of utmost importance particularly when dealing with gluten‐free dough.^26^, ^27^

Previous studies suggested that there is scanty of research emphasizing the effect of 3D printing parameters in the development of composite gluten‐free dough. Also, it is imperative to analyse the stability and structural deformation of the printed cereal‐based dough after baking, which is carried out in the present study and contributes to its novelty. The present study deals with the development of a composite dough comprising gluten‐free ingredients, including little millet, amaranth and curry leaf powder, aiming to explore its dough characteristics, printability and post baking analysis for 3D food manufacturing. The objectives of the present work are (i) to investigate the nutritional profile and viscoelastic properties of the composite dough; (ii) to optimize the 3D printing process parameters such as nozzle diameter, extrusion rate, print speed and layer height and to carry out correlation analysis and principal component analysis to theoretically validate the optimized results; and (iii) to perform the post baking evaluations in terms of printability assessments and textural analysis of the optimized samples. This study is crucial for ensuring the dough extrudability and structural integrity of the printed product and to confirm that the obtained printing parameters can effectively contribute to the development of a precise functional product.

## MATERIALS AND METHODS

### Raw materials

Little millet (*Panicum miliare*), amaranth seeds (*Amaranthus caudatus* L.), curry leaves (*Murraya koenigii*) and wheat flour (*Triticum aestivum* L.) were purchased from the local marketplace of IIT Kharagpur (West Bengal, India). After cleaning, the little millet and amaranth seeds were milled into flour and sieved under 250‐μm mesh size.^3^ The curry leaves were dried in tray dryer (S.D. Instruments & Equipments, Kolkata, India) at 40 °C and made into powder and sieved. The samples were stored in polybags of 250 gauge for further use. Additional ingredients such as sugar and vegetable oil were acquired for dough preparation.

### Nutritional profile analysis

The proximate composition of little millet flour (LMF), amaranth seed flour (ASF), curry leaf flour (CLF) and wheat flour (WF) was determined in terms of carbohydrate, fat, protein, crude fiber, ash and moisture content using AOAC procedures.^28^

### Composite flour formulation

A total of three composite flour were formulated (i.e., S1, S2 and S3) having a LMF:ASF:CLF proportion of 30:60:10, 40:50:10 and 50:40:10, (w/w), respectively. The CLF proportion was kept constant at 100 g kg^−1^ in all composite flour blends because exceeding this limit caused breakage in the 3D extruded dough and bitter taste in the baked product, which was determined through preliminary experiments.

### Rheological analysis of the composite flour

The dynamic oscillatory tests were carried out in a rheometer (MCR 52; Anton Paar, Graz, Austria) by slightly modifying the approach of Pradhan and Tripathy.^3^ The amplitude sweep test (AST) of all the composite flour blends and the wheat flour (taken as control sample) were carried out at constant frequency of 5 rad s^−1^. The linear viscoelastic regime (LVR) of all the samples, storage modulus (*G*′) and loss modulus (*G*″) were calculated as a function of shear strain. Frequency sweep test (FST) was performed with the strain maintained within the identified LVR with varying frequency from 0.1 to 100 rad s^−1^ at 25 °C.

### Dough preparation for 3D printing

The method described by Kewuyemi *et al*.^29^ was modified and followed for gluten‐free dough preparation. The quantity of sugar, vegetable oil and distilled water per 100 g of multigrain flour formulation were taken as 20 g, 12 g and 35 mL, respectively. The dry ingredients (i.e. multigrain flour and sugar) were mixed in an electric blender (500 W; Bajaj Electricals, New Delhi, India). The dry mixture was then transformed into a cohesive dough by incorporating vegetable oil and water, by using an electric dough maker (300 W; Philips India Limited, Mumbai, India). The prepared gluten‐free dough blend was kept covered in a plastic pouch for about 1 h before 3D printing for equilibration of moisture.

### 3D Model design and printing process

**Figure 1:**
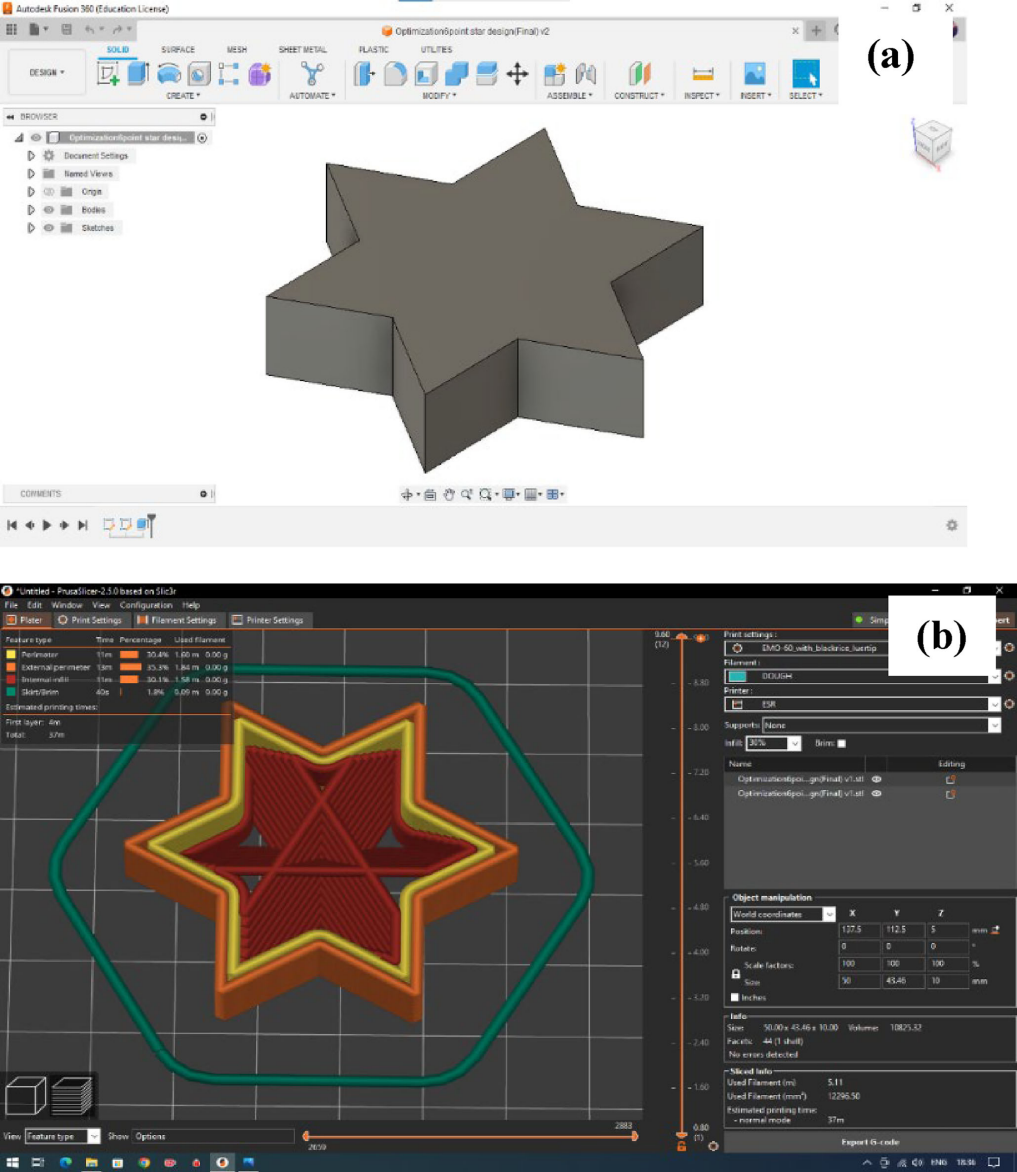
Six‐pointed star model designed for the 3D extrusion‐dough printing (a) CAD 3D model; (b) Sliced model with 30% infill.

The 3D printing of the cohesive dough was carried out in an extrusion‐based 3D printer (Engine SR, Hyrel 3D; AMS, Bangalore, India). Computer‐aided design (CAD) of a six‐pointed star geometrical shape (Fig. 1a) was designed on Autodesk Fusion 360, version 2.0.13615 (Autodesk Incorporation, San Francisco, CA, USA) and the design was saved as a stereolithography (STL) extension file. The STL file of the model was then imported into Prusa Slicer, version 2.6.0 (Prusa Research, Prague, Czech Republic), which facilitated slicing the model layer by layer for 3D printing (Fig. 1b). The fill density and fill pattern for the design was selected as 30% and ‘support cubic’, respectively. The sliced design was saved as a geometric code (G‐code) extension file. The produced G‐code was imported into Repetrel, version 4.2.597 (Hyrel 3D, Norcross, GA, USA) of the 3D printer for the dough printing. The cohesive dough was carefully loaded into the printer syringe barrels, ensuring the absence of air bubbles. The syringe was made up of food grade stainless‐steel material having a capacity of 60 mL.

### Design of the experiment

A four factor (*k*), five level (*L*) central composite design (CCD), with six centre points (*C*_0_) was selected to optimize the 3D printing process parameters using Design‐Expert 13 (Stat‐Ease Inc., Minneapolis, MN, USA). The overall number of experimental runs (*N*) was estimated using the expression given by Derringer and Suich:^30^

(1) $$N=2k\left(k-1\right)+C_{0}$$

The design included four independent factors, namely nozzle diameter (*x*_1_), extrusion rate (*x*_2_), print speed (*x*_3_) and layer height (*x*_4_). The layer height for printing was taken as percentage of the nozzle diameter. The actual and coded values of the independent factors are depicted in Table 1. The subsequent fitting of experimental data was done using the following second order polynomial function:^31^

(2) $$y_{j}=\beta_{0}+\sum_{i=1}^{3}\beta_{i}x_{i}+\sum_{i=1}^{3}\beta_{i}x_{i}^{2}+\sum \sum_{i=1}^{3}\beta_{\mathrm{ij}}x_{i}x_{j}$$

where, *x*_*i*_ and *x*_*j*_ stands for the independent factors, ranging from − α to + α, and *y*_*j*_ denotes various responses. *β*_*0*_, *β*_*i*_, *β*_*ii*_ and *β*_*ij*_ represents the regression coefficients for the mean, linear, quadratic and interaction terms, respectively. Response surface graphs were generated to visualize factor–response interactions. Moreover, the model analysis, model *F*‐value, coefficient of determination (*R* ^2^), and adjusted coefficient of determination (adj. *R* ^2^) were used to evaluate the statistical significance of the models.

**Table 1:** Independent factors associated in the experimental design

Independent variables	Terminology	Units	Coded levels (values)				
			−α	−1	0	+1	+α
Nozzle diameter	*ND* (*x*_*1*_)	mm	1.2	1.6	2.0	2.4	2.8
Extrusion rate	*ER* (*x*_*2*_)	pulse μL^−1^	60	90	120	150	180
Print speed	*PS* (*x*_*3*_)	mm s^−1^	2	4	6	8	10
Layer height	*LH* (*x*_*4*_)	%	30	45	60	75	90

The layer height is expressed as percentage of nozzle diameter.

### Printability assessment method

Printability refers to a material's ability to be extruded consistently, forming a precise shape, and maintaining dimensional stability after post‐printing operation. The geometrical print measurements of the 3D dough sample (*d*_*P*_, *A*_*P*_, *H*_*P*_, $\theta_{P}$, *V*_*P*_, $\emptyset_{P}$) were evaluated in ImageJ, version 1.8.0.345 (National Institute of Health, Bethesda, MD, USA). The target measurement values (*d*_*T*_, *A*_*T*_, *H*_*T*_, $\theta_{T}=90$, *V*_*T*_, $\emptyset_{T}=60$) were determined from the CAD 3D dough model. Here, the terms *d, A, H*, θ, *V*, ∅ signifies width, total surface area, height, slant angle, volume and adjacent angle, respectively. The responses such as printing precision (*PP*), print fidelity (*PF*), height deformation (*HD*), slant angle deviation (*SAD*), volumetric deformation (*VD*) and adjacent angle deviation (*AAD*) were calculated by using both print measurements and control measurements as represented in the formulae mentioned in Table 2. Among the above responses, *PP* and *PF* are considered as the positive index of printability, whereas *HD*, *SAD*, *VD* and *AAD* are considered as the negative index of printability. Further, the print weight (*W*) and print duration (*T*) of each sample were also noted.

**Table 2:** Printability assessment parameters (responses) and their respective goals for optimization

Responses	Formula	Goal for optimization	
Printing precision (%) (*PP*)	$\mathrm{PP}=\frac{d_{P}}{d_{T}}\times 100$	Target 100%	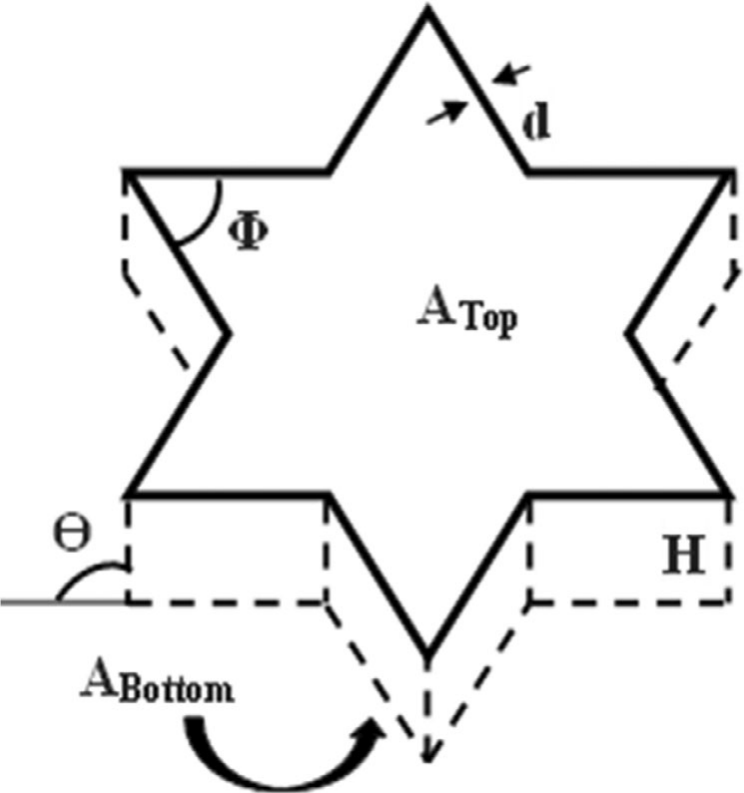
Print fidelity (%) (*PF*)	$\mathrm{PF}=\left(1-\frac{|A_{P}-A_{T}|}{A_{T}}\right)\times 100$	Maximize	
Height deformation (%) (*HD*)	$\mathrm{HD}=\frac{|H_{P}-H_{T}|}{H_{T}}\times 100$	Minimize	
Slant angle deviation (%) (*SAD*)	$\mathrm{SAD}=\frac{|\theta_{P}-90|}{90}\times 100$	Minimize	
Volumetric deformation (%) (*VD*)	$\mathrm{VD}=\frac{|V_{P}-V_{T}|}{V_{T}}\times 100$	Minimize	
Adjacent angle deviation (%) (*AAD*)	$\mathrm{AAD}=\frac{|\emptyset_{P}-60|}{60}\times 100$	Minimize	
Weight (g) (W)		In range	
Printing duration (min) (T)		Minimize	

where *d*_*p*_: printing width (mm); *d*_*T*_: target width (mm); *A*_*P*_: total surface area of the print (mm^2^), *A*_*T*_: target total surface area (mm^2^); *A*_top_: top cross‐sectional area (mm^2^); *A*_Bottom_: bottom cross‐sectional area (mm^2^); *H*_*P*_: printed height (mm); *H*_*T*_: target height (mm); $\theta_{P}$: slant angle of the print (°); *V*_*P*_: volume of the print (mm^3^); *V*_*T*_: target volume (mm^3^) and $\emptyset_{P}$: adjacent angle of the print (°).

### Multivariate analysis

Correlation analysis and principal component analysis (PCA) of the input (*ND*, *ER*, *PS* and *LH*) and response parameters (*PP, PF, HD, SAD, VD, AAD, W* and *T*) were performed using OriginPro 2023b, version 10.0.5.157 (Origin Lab Corporation, Northampton, MA, USA). Prior to performing PCA, the 30 printed samples were assigned with tags such as IE (ideal extrude), MOE (moderate over‐extrude) and HOE (high over‐extrude), based on the obtained response values and images. Then, PCA was utilized to group the samples into meaningful clusters through confidence ellipses, which reduces the dimensionality and facilitates comparison between the clusters based on their characteristics.^32^

### Selection of sample for post printing analysis

The CCD model gave ‘*n*’ number of optimized solutions with high desirability values (*D* ≤ 1), from which four sets of optimized solutions (*P*_1_, *P*_2_, *P*_3_ and *P*_4_) were selected, each with varying *ND*, *ER, PS* and *LH*. These samples were 3D printed using S1 composite flour and the control sample (*C*) was printed with wheat flour. The samples were baked at 180 °C for 15 min, and cooled to room temperature.^29^ The samples before baking were termed as *C*_*B*_, *P*_1*B*_, *P*_2*B*_, *P*_3*B*_ and *P*_4*B*_, whereas, after baking, they were termed as *C*_*A*_, *P*_1A_, *P*_2A_, *P*_3A_ and *P*_4A_. The printability assessment of the samples was undertaken before and after baking, following the same procedure as mentioned in the section above concerning the ‘Printability assessment method’.

### Texture profile analysis

The textural properties of the baked 3D printed samples were studied using a texture analyser (TexturePro CT V1.4 Bulid 17; AMETEK Brookfield, Middleboro MA, USA) following the method of Pradhan and Tripathy.^3^ It was conducted through single compression test using a flat plate probe of 30 mm in diameter and 10 mm in thickness with a 500 N load cell. The sample was allowed to 50% compression of actual thickness, with test and pretest speeds of 0.5 mm s^−1^. The maximum peak force (N) and the maximum force (N) at the first significant drop detected in the force–time graph of the compression test was assessed as the hardness and fracturability of the sample, respectively.

### Statistical analysis

Statistical analysis was carried out using IBM SPSS, version 29.0.0 (IBM Corp., Armonk, NY, USA). The variations in the chemical, printability and textural properties of the samples were analysed using a *post hoc*‐Duncan test keeping the significance level at *P* ≤ 0.05. Analysis of variance (ANOVA) was used to determine the coefficients of regression and statistical factors of the CCD model. Each experiment was conducted in triplicate and the results are reported as the mean ± SD.

## RESULTS AND DISCUSSION

### Nutritional profile of raw materials

The proximate composition of WF, LMF, ASF and CLF is given in Table 3. WF was taken as control to compare its properties with the other selected gluten‐free raw materials. Table 3 shows that LMF and CLF had nearly comparable protein content with that of WF, whereas ASF significantly exceeded WF. This was one of the reasons why ASF was chosen for composite flour formulation because of it's high protein content (16.23 ± 0.55 g) that compensates for the lack of gluten and aids in the formation of a cohesive dough.^4^ LMF was found to have high fibre (7.7 ± 0.36 g) and carbohydrate content (62.21 ± 0.46 g), making it a good base for forming a dough that can maintain structural integrity.^33^ CLF showed high fibre content (19.18 ± 0.27 g), which helps to enhance the nutritional profile of the dough. Drishya *et al*.^34^ reported that CLF incorporation can significantly enhance the protein, dietary fibre, minerals, *β*‐carotene contents and radical scavenging activity in the formulated product.

**Table 3:** Proximate composition of raw materials

Sample	Moisture (% wb)	Ash (%)	Fat (%)	Fibre (%)	Protein (%)	Carbohydrate (%)
WF	11.33 ± 0.23 ^a^	1.03 ± 0.17 ^a^	1.33 ± 0.11 ^a^	0.51 ± 0.08 ^a^	10.23 ± 0.21 ^a^	75.60 ± 0.34 ^a^
LMF	10.26 ± 0.37 ^b^	5.11 ± 0.12 ^b^	4.80 ± 0.18 ^b^	7.70 ± 0.36 ^b^	9.92 ± 0.03 ^a^	62.21 ± 0.46 ^b^
ASF	9.79 ± 0.59 ^b^	3.25 ± 0.44 ^c^	6.71 ± 0.87 ^c^	4.54 ± 0.35 ^c^	16.23 ± 0.55 ^b^	59.48 ± 0.58 ^c^
CLF	8.87 ± 0.24 ^c^	9.21 ± 0.32 ^d^	2.93 ± 0.19 ^d^	19.18 ± 0.27 ^d^	8.66 ± 0.12 ^c^	51.15 ± 0.93 ^d^

The results are expressed as the mean ± SD from triplicates (n = 3). Values in the same column with different superscript lowercase letters are significantly different at *P* ≤ 0.05.

### Viscoelastic behaviour of composite flour blends

**Figure 2:**
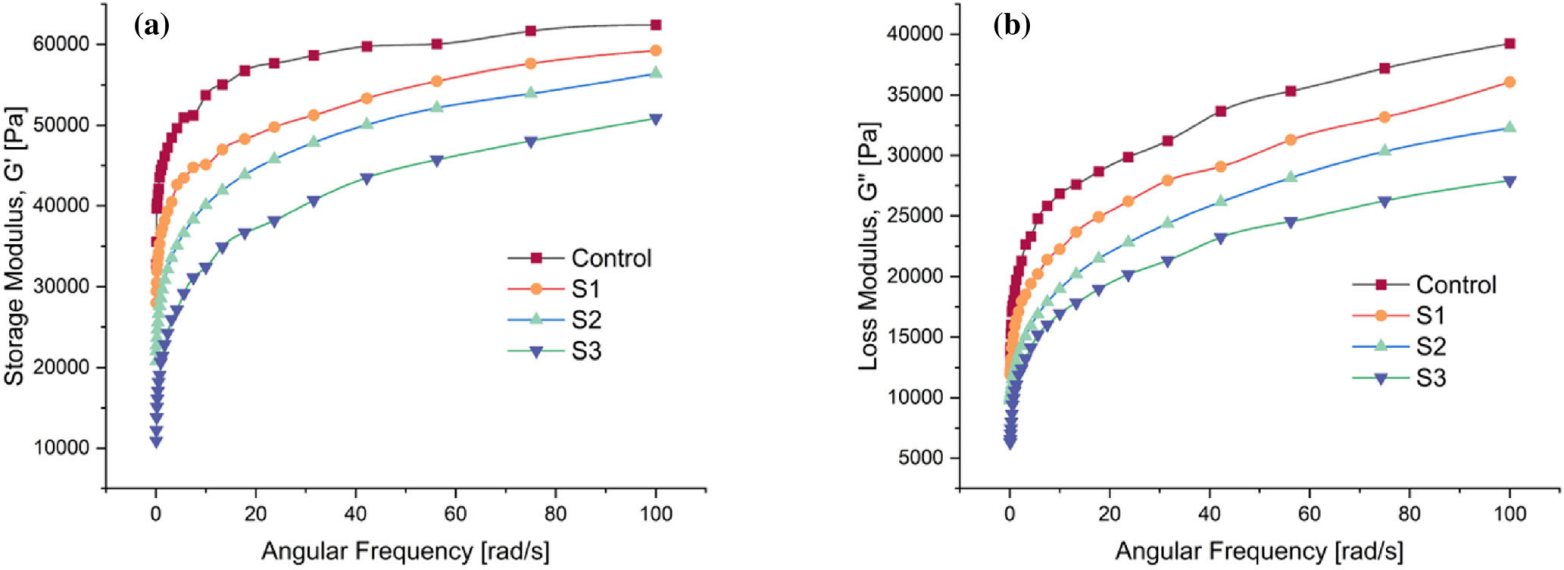
Dynamic rheology of control and composite flour samples: (a) Frequency dependence of storage modulus and (b) loss modulus.

The composite samples and the control sample were subjected to AST and FST. To compare the viscoelastic properties of the gluten‐free composite samples, WF was chosen as control as the gluten protein present in WF makes it ideal and structurally stable dough. For all samples (control, S1, S2 and S3), the LVR was found to be within 0.7% shear strain. The FST performed within LVR is seen in Fig. 2. *G*′ and *G*″ depict the elastic and viscous properties of the tested samples, respectively. Figure 2 shows that *G*′ > *G*″ for all the respective samples, illustrating the prevalence of elastic behaviour in the dough.^3^ The highest and lowest *G*′ value was observed for the control sample and S3, respectively. Similar was the case for *G*″. The S1 sample was found to have the *G*′ and *G*″ value close to the control sample, which indicated that S1 had the best viscoelastic characteristics after WF. Hence, S1 composite was selected for 3D printing because it would be more propitious for extrusion and less susceptible to breakage.

### Effect of 3D printing process parameters on printability assessment factors of the printed dough

Table 4 shows the experimental design chart of selected runs to visualize the results obtained for the measured printability variables along with the top, bottom and lateral view of the printed dough sample. The entire experimental design table for 30 experimental samples is provided in the Supporting information (Table S1). Table 5 presents the coefficients of regression and statistical factors corresponding to the input factors and responses of the CCD model, along with the ANOVA of the experimental data.

**Table 4:** Experimental design chart of selected runs to visualize the results obtained for the measured printability variables along with the top, bottom and lateral view of the printed dough sample

Run	Print variables *ND*:*ER*:*PS*:*LH*	Printability variables (responses)									Captured images		
		*PP* (%)	*PF* (%)	*HD* (%)	*SAD* (%)	*VD* (%)	*AAD* (%)	*W* (g)	*T* (min)	Tags	Top layer view	Bottom layer view	Lateral view
5	2.4:150:4:75	164.67	64.27	12.56	12.34	78.59	53.80	19.68	11.20	HOE	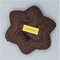	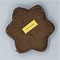	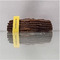
7	2:120:6:60	127.20	97.80	2.72	2.66	16.89	27.39	10.82	11.22	IE	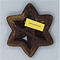	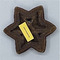	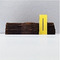
15	2.4:150:4:45	178.04	51.64	10.25	23.22	81.27	68.65	18.90	16.28	HOE	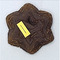	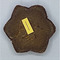	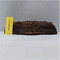
18	1.6:90:4:75	97.44	95.29	4.76	4.87	14.40	9.06	7.98	16.58	IE	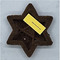	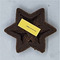	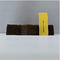
23	2:120:6:60	128.30	95.43	2.91	2.61	18.90	28.43	11.98	10.90	MOE	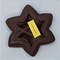	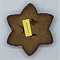	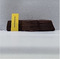

Units of the print variables are *ND* = mm; *ER* = pulse μL^−1^; *PS* = mm s^−1^; *LH* = % of nozzle diameter.

**Table 5:** Regression coefficients and statistical factors for the experimental design model

Coefficients	*PP*	*PF*	*HD*	*SAD*	*VD*	*AAD*	*W*	*T*
*β*_0_	126.12	96.82	2.94	2.49	17.8	27.21	11.61	10.97
*β*_1_	13.46^a^	−5.31^a^	3.68^a^	1.8^a^	12.8^a^	10.05^a^	2.01^a^	−3.07^a^
*β*_2_	19.74^a^	−11.63^a^	1.43^a^	3.79^a^	18.09^a^	10.36^a^	2.88^a^	−0.141^NS^
*β*_3_	−6.79^a^	1.31^b^	0.314^c^	−0.278^NS^	−1.23^NS^	−0.371^NS^	0.055^NS^	−4.78^a^
*β*_4_	3.68^a^	2.08^a^	1.16^a^	−0.505^b^	−3.43^a^	−6.98^a^	−0.402^a^	−3.17^a^
*β*_1_ *β*_2_	10.99^a^	−6.14^a^	−0.053^NS^	2.29^a^	5.94^a^	2.02^b^	1.21^a^	0.013^NS^
*β*_1_ *β*_3_	−1.63^NS^	0.474^NS^	0.736^a^	0.061^NS^	−2.01^b^	−1.46^b^	−0.147^NS^	0.990^a^
*β*_*1*_ *β*_*4*_	3.76^b^	1.3^c^	−0.438^b^	−2.37^a^	0.721^NS^	0.130^NS^	0.309^c^	1.17^a^
*β*_2_ *β*_3_	−4.73^a^	0.797^NS^	0.103^NS^	−1.57^a^	−1.09^NS^	−2.45^a^	−0.327^b^	−0.030^NS^
*β*_2_ *β*_4_	−6.3^a^	2.19^a^	−0.387^c^	−1.05^a^	−1.2^NS^	−1.02^NS^	0.139^NS^	−0.021^NS^
*β*_3_ *β*_4_	7.11^a^	0.07^NS^	−0.872^a^	0.911^a^	−1.82^c^	−2.06^b^	−0.306^c^	0.942^a^
*β*_11_	−5.38^a^	−2.9^a^	2.06^a^	2.16^a^	7.9^a^	2.05^a^	0.672^a^	0.434^c^
*β*_22_	1.02^NS^	−5.41^a^	0.879^a^	1.98^a^	8.17^a^	5.31^a^	−0.207^NS^	−0.182^NS^
*β*_33_	−4.04^a^	−0.856^NS^	0.587^a^	1.56^a^	1.27^c^	−0.698^NS^	−0.030^NS^	1.86^a^
*β*_44_	−6.45^a^	−2.47^a^	0.862^a^	0.125^NS^	5.25^a^	2.42^a^	0.300^b^	0.882^a^
Model (*F*‐value)	17.83^a^	34.87^a^	52.57^a^	71.54^a^	62.07^a^	26.71^a^	10.28^a^	19.90^a^
*R* ^2^	0.9795	0.9819	0.9835	0.9862	0.9874	0.9819	0.9824	0.9822
Adj. *R* ^2^	0.9604	0.9650	0.9680	0.9734	0.9757	0.9650	0.9659	0.9655
CV (%)	4.76	3.05	9.37	9.94	7.37	8.77	5.28	8.95

*β*_1_, *β*_2_, *β*_3_ and *β*_4_ represents the coefficients of *ND*, *ER*, *PS* and *LH*, respectively, whereas *β*_0_ is the intercept of the eqs. NS: insignificant (*P* > 0.1). ^a^Highly significant (*P* < 0.01). ^b^Moderately significant (0.01 < *P* < 0.05). ^c^Significant (0.05 < *P* < 0.1).

#### Print precision and print fidelity

The print precision (*PP*) and print fidelity (*PF*) of the samples varied from 74% to 178.04% and 51.64% to 99.84%, respectively. The results of the regression analysis showed that the linear factors (*β*_1_, *β*_2_, *β*_3_ and *β*_4_) of nozzle diameter (*ND*), extrusion rate (*ER*), print speed (*PS*) and layer height (*LH*) exhibited a strongly significant effect (*P* < 0.01) on both *PP* and *PF*. The quadratic equations representing the effects of the 3D printing process parameters on *PP* and *PF* are expressed in Eqns (3) and (4), respectively.

(3) $$\mathrm{PP}=\begin{array}{l} 126.12+13.46x_{1}+19.74x_{2}-6.79x_{3}+3.68x_{4}\\ +\;10.99x_{1}x_{2}+3.76x_{1}x_{4}-4.73x_{2}x_{3}-6.3x_{2}x_{4}\\ +\;7.11x_{3}x_{4}-5.38x_{1}^{2}-4.04x_{3}^{2}-6.45x_{4}^{2} \end{array}$$

(4) $$\mathrm{PF}=\begin{array}{l} 96.82-5.31x_{1}-11.63x_{2}+1.31x_{3}+2.08x_{4}-6.14x_{1}x_{2}\\ +\;1.3x_{1}x_{4}+2.19x_{2}x_{4}-2.9x_{1}^{2}-5.41x_{2}^{2}-2.47x_{4}^{2} \end{array}$$

**Figure 3:**
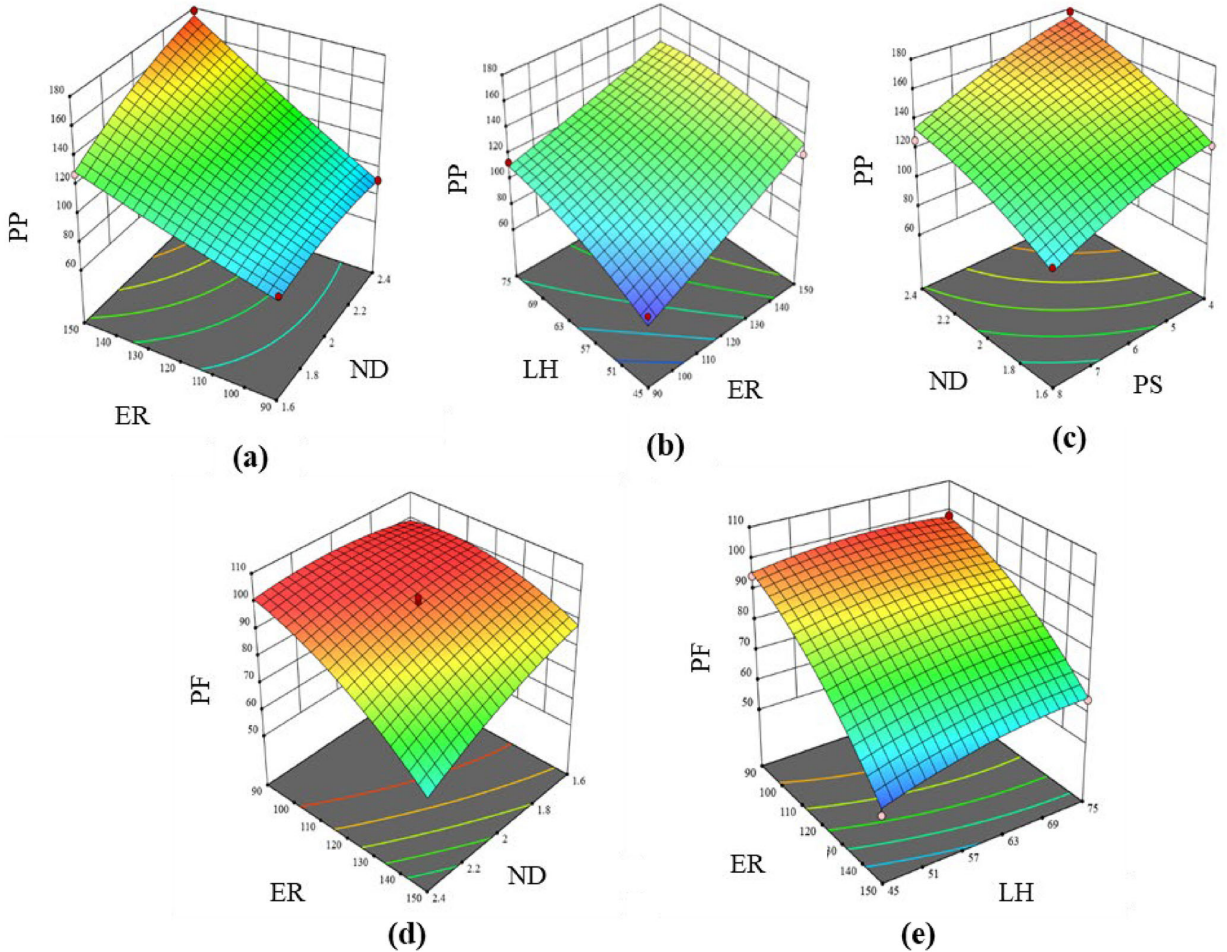
3D interaction plots showing the effect of (a) *ND* and *ER* on *PP*; (b) *ER* and *LH* on *PP*; (c) *ND* and *PS* on *PP*; (d) *ND* and *ER* on *PF*; (e) *ER* and *LH* on *PF*.

Figure 3 shows the effect of various parameters such as *ND*, *ER*, *PS* and *LH* on *PP* and *PF*. Print precision refers to the degree of accuracy and consistency with which the 3D printer can produce physical objects that closely match the specifications of the designed CAD 3D model. To achieve a target of 100% for *PP*, it is expected that the diameter of the extruded dough filament matches with that of the nozzle, assuming no shrinkage or swelling.^35^ Maximum deviation of *PP* (from 100%) was observed when both *ND* and *E*R were at their lowest and highest values (Fig. 3a). The case was the same for *ER* and *LH* (Fig. 3b). *PS* had a negative correlation with *PP*, as observed from the response surface plot (Fig. 3c). Similar findings were reported in a study where the print precision of the egg yolk material was found to decrease when the *PS* and *ND* were increased beyond 800 mm min^−1^ and 0.84 mm, respectively.^36^ *PF* refers to the ability of a 3D printer to accurately reproduce the desired shape and size of the product, with minimal distortion and errors as calculated in terms of its total surface area.^37^ In this study, *PF* decreased with an increase in *ND* (Fig. 3d) because using a larger nozzle diameter resulted in uncontrolled material flow. Furthermore, the *PF* significantly decreased at higher extrusion rates beyond 120 pulse μL^−1^ (Fig. 3e). At too high *ER*, the printer may struggle to accurately control the material flow resulting in errors, which can lead to defects in the print.^38^

#### Print geometric deviations

Print geometric deviations such as volumetric deformations and angle deformations are significant factors in 3D food printing because they impact the final product's shape, texture and overall quality.^39^ The variation of *HD*, *SAD*, *AAD* and *VD* was found to be 0.36–18.56%, 0.18–23.22%, 9.06–68.64% and 12.54–86.13%, respectively. *HD* refers to the variation in the height of the printed product with respect to the target geometry. On the other hand, *SAD* and *AAD* refer to the deviation in printed angles from their intended values. All these responses attributed to the overall percent dimensional change (*VD*) in the intricate structure of the print with respect to the target geometry. The model quadratic equations representing the effects of the 3D printing parameters on *HD*, *SAD*, *AAD* and *VD* are expressed in Eqns ((5), (6), (7), (8)).

(5) $$\mathrm{HD}=\begin{array}{l} 2.94+3.68x_{1}+1.43x_{2}+0.314x_{3}+1.16x_{4}+0.736x_{1}x_{3}\\ -\;0.438x_{1}x_{4}-0.387x_{2}x_{4}-0.872x_{3}x_{4}+2.06x_{1}^{2}\\ +\;0.879x_{2}^{2}+0.587x_{3}^{2}+0.862x_{4}^{2} \end{array}$$

(6) $$\mathrm{SAD}=\begin{array}{l} 2.49+1.8x_{1}+3.79x_{2}-0.505x_{4}+2.29x_{1}x_{2}-2.37x_{1}x_{4}\\ -\;1.57x_{2}x_{3}-1.05x_{2}x_{4}+0.911x_{3}x_{4}+2.16x_{1}^{2}+1.98x_{2}^{2}\\ +\;1.56x_{3}^{2} \end{array}$$

(7) $$\mathrm{AAD}=\begin{array}{l} 27.21+10.05x_{1}+10.36x_{2}-6.98x_{4}+2.02x_{1}x_{2}\\ -\;1.46x_{1}x_{3}-2.45x_{2}x_{3}-2.06x_{3}x_{4}+2.05x_{1}^{2}+5.31x_{2}^{2}\\ +\;2.42x_{4}^{2} \end{array}$$

(8) $$\mathrm{VD}=\begin{array}{l} 17.8+12.8x_{1}+18.07x_{2}-3.43x_{4}+5.94x_{1}x_{2}-2.01x_{1}x_{3}\\ -\;1.82x_{3}x_{4}+7.9x_{1}^{2}+8.17x_{2}^{2}+1.27x_{3}^{2}+5.25x_{4}^{2} \end{array}$$

**Figure 4:**
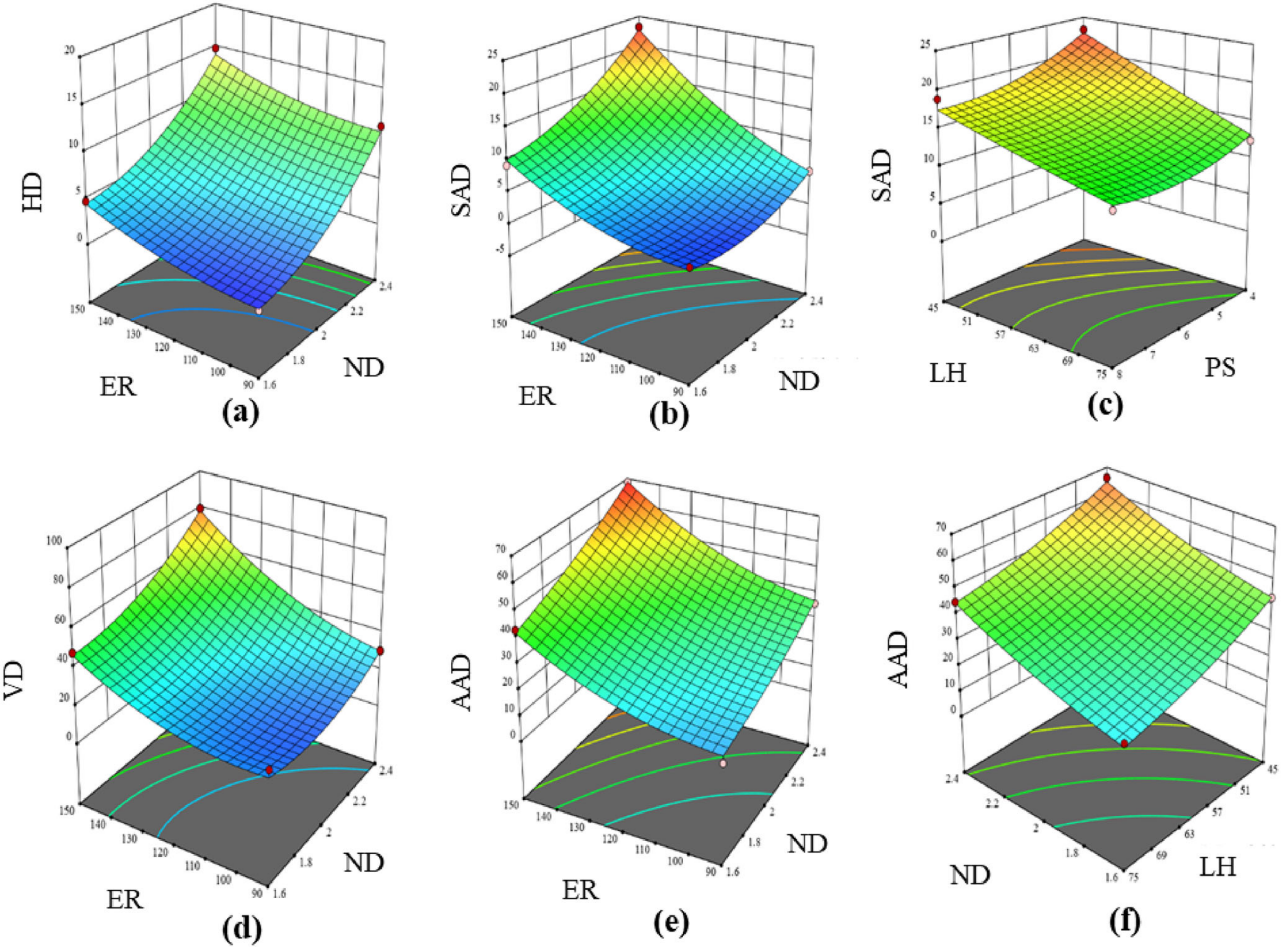
3D interaction plots showing the effect of (a) *ND* and *ER* on *HD*; (b) *ND* and *ER* on *SAD*; (c) *PS* and *LH* on *SAD*; (d) *ND* and *ER* on *VD*; (e) *ND* and *ER* on *AAD*; (f) *ND* and *LH* on *AAD*.

Figure 4 shows the influence of *ND*, *ER*, *PS* and *LH* on *HD*, *SAD*, *AAD* and *VD* of the printed structure. All forms of print geometric deviations were found to be amplified when printing with *ND* of 2.4 and 2.8 mm as a result of the uncontrolled material flow. The case was the same for the effect of *ER* on the individual deviations. *SAD* and *AAD* decreased with an increasing layer height up to 75% of *ND* for dough printing (Fig. 4c,f). However, a further increase in layer height significantly intensified the geometric deviations resulting in initiation of post printing collapse because of gravity.^40^ An increase in printing speed resulted in a decrement in *VD*, *SAD* and *AAD*. The print speed was not raised beyond 10 mm s^−1^ to ensure smooth extrudability of the material.

#### Print weight and printing duration

Print weight (*W*) and print duration (*T*) are quite interrelated to each other because increasing the *W* can also lead to longer *T* and higher material usage, leading to an increase in the cost of 3D printing. Accordingly, *W* and *T* are two key responses that are majorly influenced by the factors such as *PS* and *LH*. In this study, *W* and *T* for the composite dough varied from 4.26 to 19.68 g and 5.82 to 30.75 min. The regression model as described in Eqns (9) and (10) showed a significant (*P* < 0.05) linear, interactive and quadratic effect of the independent printing factors on the print weight and printing duration.

(9) $$W=\begin{array}{l} 11.61+2.01x_{1}+2.88x_{2}-0.402x_{4}+1.21x_{1}x_{2}\\ +\;0.309x_{1}x_{4}-0.327x_{2}x_{3}-0.306x_{3}x_{4}+0.672x_{1}^{2}+0.3x_{4}^{2} \end{array}$$

(10) $$T=\begin{array}{l} 10.97-3.07x_{1}-4.78x_{3}-3.17x_{4}+0.99x_{1}x_{3}+1.17x_{1}x_{4}\\ +\;0.942x_{3}x_{4}+0.434x_{1}^{2}+1.86x_{3}^{2}+0.882x_{4}^{2} \end{array}$$

**Figure 5:**
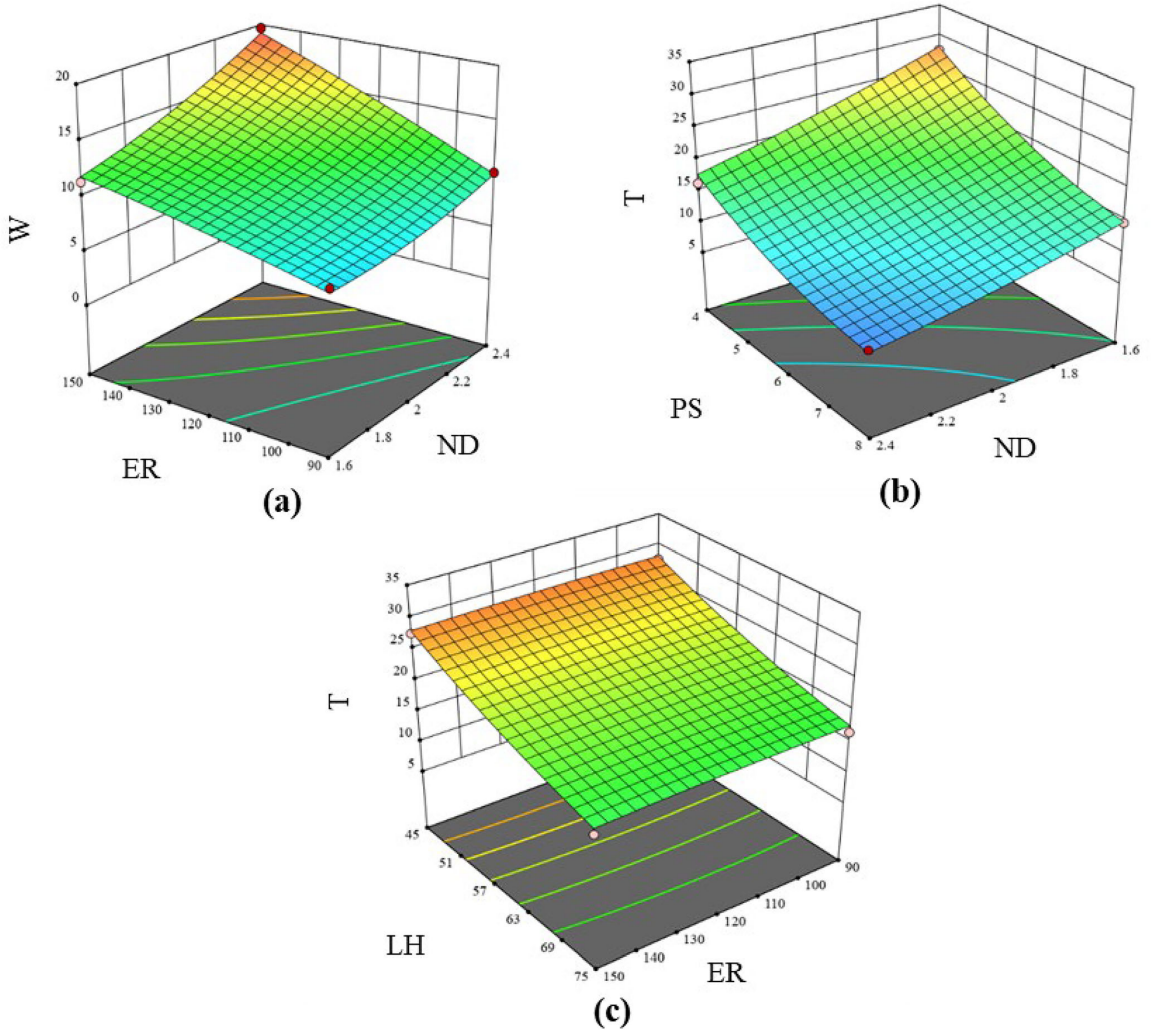
3D interaction plots showing the effect of (a) *ND* and *ER* on *W*; (b) *ND* and *PS* on *T*; (c) *ER* and *LH* on *T*.

*W* showed a significant increasing value with the increase in *ND* and *ER* (Fig. 5a) because, with increased *ND*, more material was extruded per unit length of the printed path. An increased *ER* also allows for a greater amount of material deposition within the same timeframe, contributing to the rise in overall *W*. Moreover, a linear reduction in *T* was observed for the composite dough with increasing *ND*, and quite obviously, the *T* executed a significant inverse relation with *PS* (Fig. 5b). *T* was observed to reduce at higher *LH* (Fig. 5c). This might be a result of the smaller layer height making a greater number of print layers that would require the extruder to perform more times with respect to priming and unpriming. This leads to some extra time incurred during layer transition.

### Multivariate analysis

#### Correlation analysis

**Figure 6:**
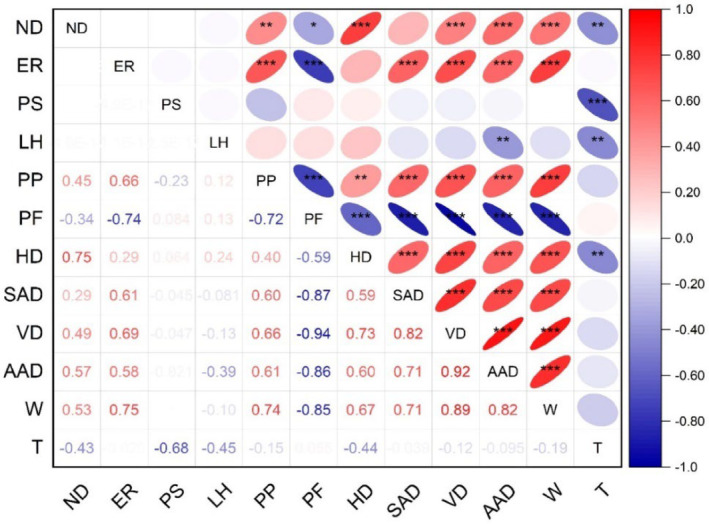
Correlation matrix displaying Pearson correlation coefficients among input variables and responses. (statistically significance difference between the parameters: *P ≤ 0.1; **P ≤ 0.05; ***P ≤ 0.01).

Figure 6 presents the correlation matrix containing Pearson's correlation coefficient (*r*). The *r* values assess the degree of association between various pairs of variables. Correlation analysis of the 3D printing input factors and the output response data displayed highest correlation of *ND* with *HD* (0.75), followed by *AAD* (0.57) and *W* (0.53). Moreover, negative correlations were shown by *ER* with *PF* (*r* = −0.74, *P* ≤ 0.05) and *PS* with *T* (*r* = −0.68, *P* ≤ 0.05). *PF* exhibited a strong negative relationship with *SAD* (*r* = −0.87, *P* ≤ 0.05), *VD* (*r* = −0.94, *P* ≤ 0.05), *AAD* (*r* = −0.86, *P* ≤ 0.05) and *W* (*r* = −0.85, *P* ≤ 0.05). This is attributed to the fact that *PF* encompasses factors such as dimensional accuracy, surface finish and overall quality of the printed object, which are negatively influenced by geometric deformations.^24^ Significant positive correlations were observed among *SAD* and *VD* (*r* = 0.82, *P* ≤ 0.05), *VD* and *AAD* (*r* = 0.92, *P* ≤ 0.05), *VD* and *W* (*r* = 0.89, *P* ≤ 0.05), and *AAD* and *W* (*r* = 0.82, *P* ≤ 0.05). This was in accordance with Kadival *et al*.^24^ stating that the larger and heavier prints often require more infill support to maintain their shape during printing. If these infill supports were not adequately positioned, they can contribute to greater geometric deformations or sagging of the printed object. Furthermore, a strong correlation was also observed between *ER* and *W* (*r* = 0.75, *P* ≤ 0.05). This can be attributed to the increase in flow level, resulting in a surge of extrusion dough material, consequently leading to a hike in the total volume and weight of the samples. This observation aligns with the results reported by Derossi *et al*.^41^ in their study on the development of 3D printed fruit‐based snacks especially for children.

#### PCA with confidence ellipse

**Figure 7:**
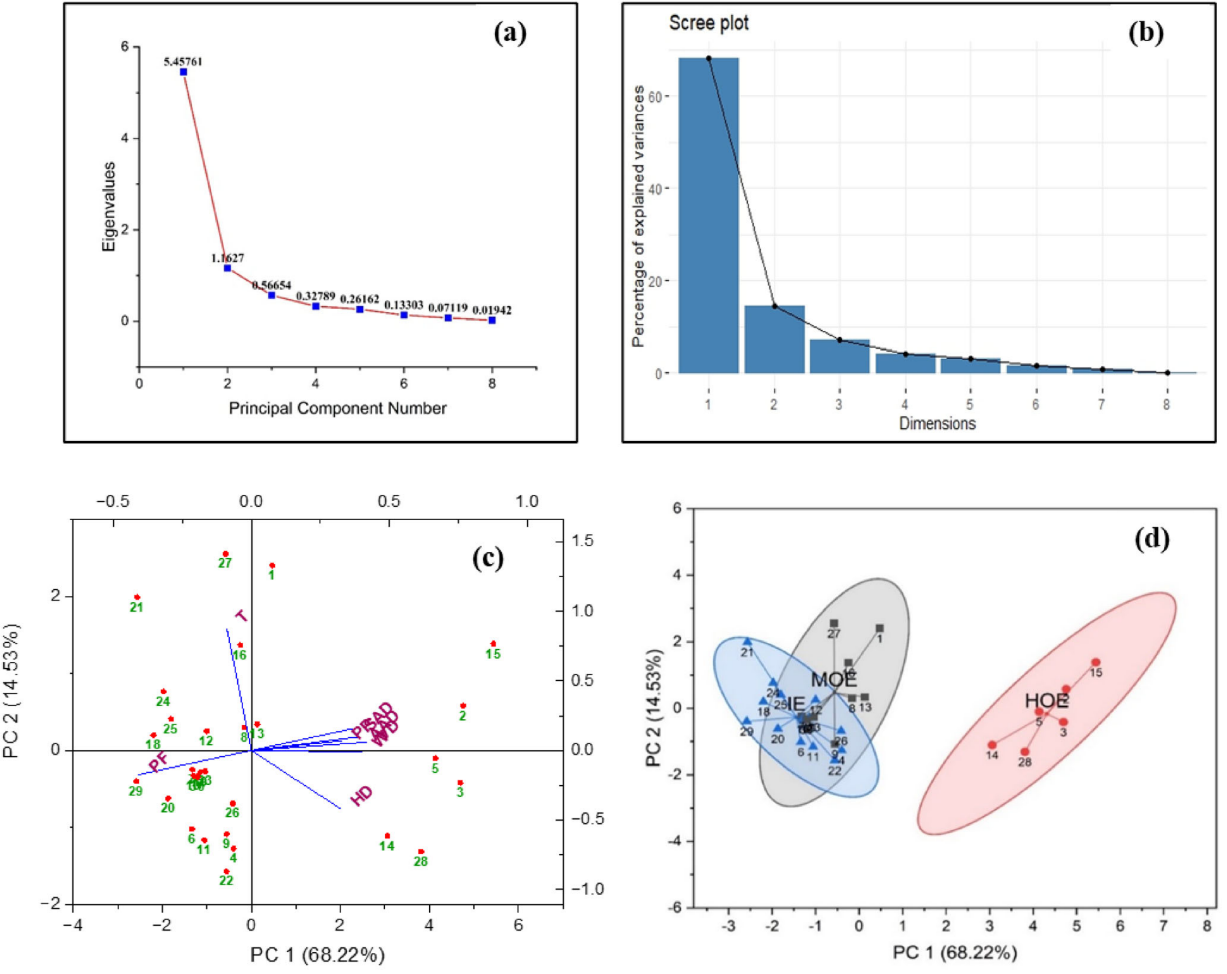
Principal component analysis of the responses: (a) Eigen value *versus* PC number scree plot; (b) Variance ratio scree plot; (c) PCA biplot representing the distribution of samples and response variables; (d) PCA with confidence ellipse. (The numbers 1, 2, 3 … 30 in (c) and (d) denote the individual samples according to the experimental runs).

PCA was performed for the responses of the 30 experimental run samples. Figure 7(a,b) demonstrates the scree plots depicting eigenvalues and the percentage of variance respectively, which corresponds to the responses (*PP*, *PF*, *HD*, *SAD*, *VD*, *AAD*, *W* and *T*). It is notable that the first two components exhibited eigenvalues greater than 1 (first component: PC1 = 5.457; second component: PC2 = 1.162). This condition follows the Kaiser–Guttman criterion, stipulating that only the components with eigenvalues exceeding 1 should be retained for further analysis.^42^ Adhering to this criterion, the primary two principal components (PC1 and PC2) were chosen to best explain the observed variability.^43^ PC1 and PC2 independently accounted for 68.22% and 14.53% of the total variance, respectively. Figure 7(c) displays a PCA bi‐plot representing the correlation of response patterns and the connections between different 3D printed samples in a multi‐dimensional space. It can be observed that majority of the responses (*PP*, *SAD*, *AAD*, *VD* and *W*) were positively correlated with both PC1 and PC2, whereas *PF* was found to be negatively correlated. Moreover, a similar contradictory correlation was also observed between *T* and *HD*. *T* was found to be negatively correlated with PC1 and positively with PC2. On the other hand, *HD* exhibited positive correlation with PC1 and negative with PC2, implying that, with printing time enhancement, the height deformation was inversely influenced. This was in agreement with the findings of Nijdam *et al*.,^44^ who stated that molten food ink filaments need adequate time to solidify after been extruded. They gradually retain their structure and sustain the layers printed on top of them undergoing minimal geometric deviations. Figure 7(d) illustrates the PCA bi‐plot with confidence ellipse, where a total of three optimal clusters (ellipses) were obtained. The clusters were designated as IE (ideal‐extrude), MOE (moderate over‐extrude) and HOE (high over‐extrude), respectively. The IE cluster (with experimental runs of 4, 6, 7, 10, 11, 12, 18, 20, 21, 22, 24, 25 and 29) had all the samples with ‘excellent print quality’ that possessed fidelity nearer to 100% with minimal geometric deviations achieved at adequately considerable print duration. The MOE cluster (1, 8, 9, 13, 16, 17, 19, 23, 26, 27 and 30) comprised of all the samples with ‘moderate print quality’. HOE cluster (2, 3, 5, 14, 15 and 28) encompassed all the samples with ‘poor print quality’ exhibiting much higher geometric deviations with poor fidelity. It can be observed that clusters IE and MOE were closely aligned because of overlapping outliers, suggesting similarity between data points.^32^

### Optimized values and selection of most desired parameters

Optimization was achieved by maximizing *PF*, targeting *PP* to 100%, minimizing the geometric deviations (*HD*, *SAD*, *AAD*, *VD*), as well as *T* and fixing *W* in a range (10–14 g) as mentioned in Table 2. As described above in the section on ‘Selection of sample for post printing analysis’, a total of four optimal solutions, *P*_1_, *P*_2_, *P*_3_ and *P*_4_, were selected with high desirability values i.e., 0.883, 0.866, 0.860 and 0.855, respectively. The optimal values of *ND*:*ER*:*PS*:*LH* for the samples *P*_1_, *P*_2_, *P*_3_ and *P*_4_ were 1.8:115:8:70, 1.6:115:7.8:65, 2:100:7.8:55 and 2:105:6.2:75 (mm: pulse μL^−1^: mm s^−1^: %), respectively. The *P*_1_, *P*_2_, *P*_3_ and *P*_4_ samples were printed using the S1 composite flour and the control sample (*C*) were printed with WF using the *P*_1_ solution parameters (*ND*:*ER*:*PS*:*LH* – 1.8:115:8:70) because it had the highest desirability value (0.883).

### Effect of baking on printability properties

The printability assessment factors of the printed samples before baking (*C*_*B*_, *P*_1*B*_, *P*_2*B*_, *P*_3*B*_ and *P*_4*B*_) and after baking (*C*_*A*_, *P*_1*A*_, *P*_2*A*_, *P*_3*A*_ and *P*_4*A*_) are reported in the Table 6. It was observed that baking led to decrement in *PF*, whereas there was an increase in *HD*, *SAD*, *VD* and *AAD*. A slight expansion of the 3D printed structure was detected after baking, which could be a result of the release of gases (e.g. carbon dioxide or steam) from leavening agents or moisture evaporation, causing the dough to puff up.^45^ Among the baked samples, *C*_*A*_ showed the highest *PP* (98.56%) and *PF* (93.93%); and showed the lowest geometric deviation: *HD* (3.79%), *SAD* (1.05%), *VD* (13.94%) and *AAD* (14.78%). The dough sample with WF has better 3D printability compared to millet‐based flour because wheat flour contains gluten, a protein that provides elasticity, cohesion and strength to the dough, allowing it to hold its shape more effectively during and after printing.^17^ The closest sample to *C*_*A*_ in terms of printability was found to be *P*_1*A*_ with *PP*, *PF*, *HD*, *SAD*, *VD* and *AAD* at 97.81%, 85.66%, 9.83%, 0.55%, 14.40% and 18.42%, respectively, and also the printing time was lowest for *P*_1*A*_ (8.68 min).

**Table 6:** Printability assessment of the samples before and after baking

Sample	*PP*	*PF*	*HD*	*SAD*	*VD*	*AAD*	*T*	Captured images	
	(%)	(%)	(%)	(%)	(%)	(%)	(min)	Top view	Lateral view
Before baking									
*C*_*B*_	100.31 ± 0.61 ^b^	94.40 ± 1.31 ^f^	5.61 ± 0.18 ^b^	0.49 ± 0.12 ^a^	12.32 ± 0.66 ^a^	15.50 ± 0.87 ^a^	8.68	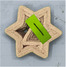	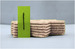
*P*_1*B*_	99.21 ± 0.49 ^b^	90.48 ± 1.98 ^e^	1.68 ± 0.17 ^a^	0.31 ± 0.11 ^a^	13.48 ± 0.64 ^a^	17.28 ± 0.83 ^ab^	8.68	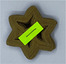	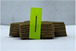
*P*_2*B*_	97.50 ± 1.20 ^ab^	82.31 ± 1.56 ^cd^	19.96 ± 1.66 ^d^	1.61 ± 0.36 ^b^	16.84 ± 1.27 ^b^	22.93 ± 0.77 ^c^	8.73	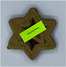	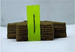
*P*_3*B*_	109.34 ± 2.67 ^c^	76.52 ± 3.31 ^b^	24.41 ± 0.81 ^e^	2.66 ± 0.07 ^c^	25.38 ± 1.14 ^c^	27.51 ± 0.95 ^de^	8.82	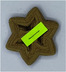	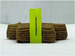
*P*_4*B*_	114.01 ± 1.61 ^d^	80.41 ± 1.19 ^c^	36.95 ± 0.75 ^f^	3.36 ± 0.58 ^c^	41.45 ± 1.40 ^e^	27.50 ± 0.88 ^de^	16.37	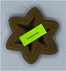	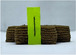
After baking									
*C*_*A*_	97.93 ± 0.88 ^ab^	93.27 ± 0.92 ^ef^	3.40 ± 0.55 ^ab^	1.25 ± 0.28 ^ab^	14.66 ± 1.02 ^ab^	15.82 ± 1.48 ^a^	8.68	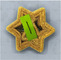	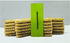
*P*_1*A*_	97.29 ± 0.73 ^ab^	84.92 ± 1.04 ^d^	10.40 ± 0.81 ^c^	0.66 ± 0.16 ^ab^	15.26 ± 1.21 ^ab^	19.15 ± 1.03 ^b^	8.68	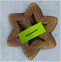	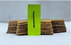
*P*_2*A*_	94.72 ± 0.83 ^a^	74.93 ± 1.40 ^b^	24.21 ± 0.74 ^e^	3.13 ± 0.47 ^c^	24.28 ± 1.49 ^c^	25.61 ± 1.94 ^d^	8.73	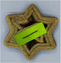	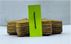
*P*_3*A*_	111.59 ± 3.31 ^cd^	69.42 ± 1.12 ^a^	35.50 ± 0.89 ^f^	3.45 ± 0.61 ^c^	29.29 ± 0.54 ^d^	28.46 ± 0.94 ^e^	8.82	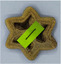	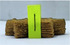
*P*_4*A*_	118.87 ± 2.29 ^e^	76.59 ± 1.23 ^b^	43.40 ± 2.16 ^g^	10.47 ± 0.72 ^d^	43.92 ± 2.57 ^e^	31.52 ± 1.32 ^f^	16.37	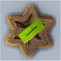	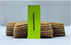

The results are expressed as the mean ± SD from triplicates (*n* = 3). Values in the same column with different superscript lowercase letters are significantly different at *P* ≤ 0.05. Subscript ‘B’ stands for ‘before’ and ‘A’ stands for ‘after’. C, Control; P, printed sample.

### Textural properties of baked samples

**Figure 8:**
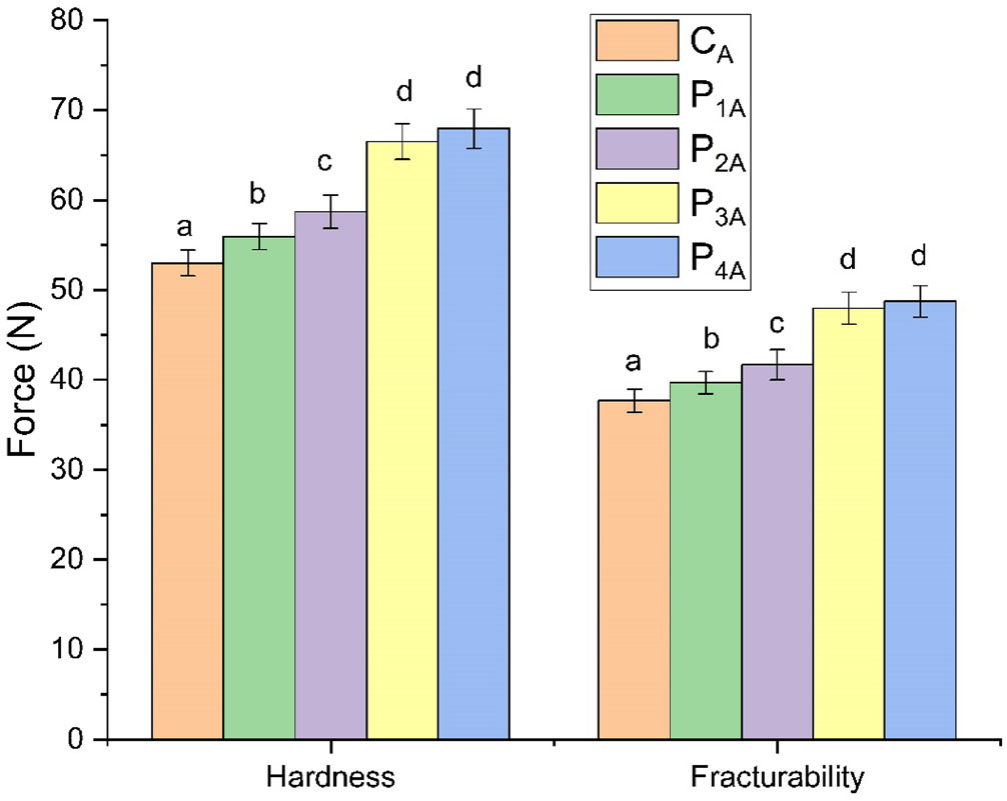
Variability of hardness and fracturability of baked samples. Samples with different lowercase letters above the bars are significantly different at *p* ≤ 0.05.

The textural characteristics of the baked six‐point star dough is illustrated in the Fig. 8. Among all the samples, the control sample was found to be lowest in hardness and fraturability values (i.e. 52.99 and 37.68 N, respectively). The hardness and fracturability of the composite samples were found to be up to 29.35% and 30.84% more than the control sample, respectively. The *P*_4*A*_ sample printed with higher *ND* (2 mm) and low *PS* (6.2 mm s^−1^) showed the highest hardness and fracturability values (i.e. 67.95 N and 48.74 N, respectively), which might be due to the fact that more amount of sample was deposited in less amount of time, leading to increment in the compactness of the printed product. No significant difference (*P* ≤ 0.05) was observed between *P*_4*A*_ and *P*_3*A*_. Among the composite samples, the closest sample to that of control (*C*_*A*_) in terms of low hardness (55.95 N) and fracturability (39.66 N) was found to be the *P*_1*A*_ sample.

## CONCLUSIONS

The selection of a gluten‐free composite dough and the effect of 3D printing process parameters on its printability was assessed through rheological analysis, response surface optimization, multivariate study and post baking analysis. LMF, ASF and CLF showed significantly high (*P* ≤ 0.05) fibre content than the control WF. The S1 composite (30% LMF, 60% ASF, 10% CLF) was found to have the nearest *G*′ and *G*″ values to WF; hence, it was selected for 3D printing. Correlation analysis of the input parameters and responses showed that the geometric deviation factors (*HD*, *SAD*, *AAD* and *VD*) exhibited positive correlation with *ND* (*r* = 0.29 to 0.75; *P* ≤ 0.01) and *ER* (*r* = 0.29–0.69; *P* ≤ 0.01) and negative correlations with *PF* (*r* = −0.59 to −0.94; *P* ≤ 0.01). PCA with PC1 and PC2 contributing 68.22% and 14.53% respectively, with total variability of 82.75% classified the experimental samples into three groups, namely, over‐extrude, moderate‐extrude and ideal extrude, through image analysis. The best sample after baking in terms of printability was found to be *P*_1*A*_ with high *PP* (97.81%) and *PF* (85.66%) and low *HD* (9.83%), *SAD* (0.55%), *VD* (14.40%) and *AAD* (18.42%). *P*_1*A*_ also exhibited lowest printing time (8.68 min) and significantly low (*P* ≤ 0.05) hardness (55.95 N) and fracturabililty (39.66 N). Hence, the printing parameters of *P*_1*A*_ (i.e. 1.8 mm of *ND*, 115 pulse μL^−1^ of *ER*, 8 mm s^−1^ of *PS* and 1.35 mm of *LH*) was considered as the most desirable optimized sample, which also fell under the PCA ideal extrude group. The proposed values of 3D printing process parameters can be used as standards to print high fibre gluten free dough with variable formulations but with similar consistency. This suggests a departure from traditional processing methods towards more advanced manufacturing technique, given the increasing demand for gluten‐free substitute in the market. Future research on 3D‐printed composite product should focus on detailed sensory analysis to enhance consumer acceptability, shelf life and stability studies and also ensure the preservation of quality and safety of the final product during storage. Moreover, the findings of this research could pave the way for its widespread commercial application, enabling the production of customizable, nutritionally enhanced gluten‐free products with improved texture, structure and efficiency in large‐scale food manufacturing.

## Supporting information


**Table S1.** Experimental design chart with the results obtained for the measured printability variable along with the top, bottom and lateral view of the printed dough sample.

## Data Availability

The data that support the findings of this study are available from the corresponding author upon reasonable request.
