# Supplementary material for: A multivariate study on the effect of 3D printing parameters on the printability of gluten‐free composite dough
Source: J Sci Food Agric. 2024 Nov 28;105(4):2578–93. doi: 10.1002/jsfa.14031 (PMC11824920; doi:10.1002/jsfa.14031)
Supplement: Supplementary file 1 — Table S1. Experimental design chart with the results obtained for the measured printability variable along with the top, bottom and lateral view of the printed dough sample. [file JSFA-105-2578-s001.docx]

**Table S1:** Experimental design chart with the results obtained for the measured printability variable along with the top, bottom and lateral view of the printed dough sample

| **Run** | **Print variables**  **ND:ER:PS:LH** | **Printability Variables (Responses)** | | | | | | | |  | **Captured images** | | |
| --- | --- | --- | --- | --- | --- | --- | --- | --- | --- | --- | --- | --- | --- |
|  |  | **PP (%)** | **PF (%)** | **HD (%)** | **SAD (%)** | **VD (%)** | **AAD (%)** | **W (g)** | **T (min)** | **Tag** | **Top layer view** | **Bottom layer view** | **Lateral view** |
| 1 | 1.6:150:4:45 | 127.94 | 81.68 | 3.76 | 9.13 | 44.79 | 43.04 | 12.55 | 27.57 | MOE | 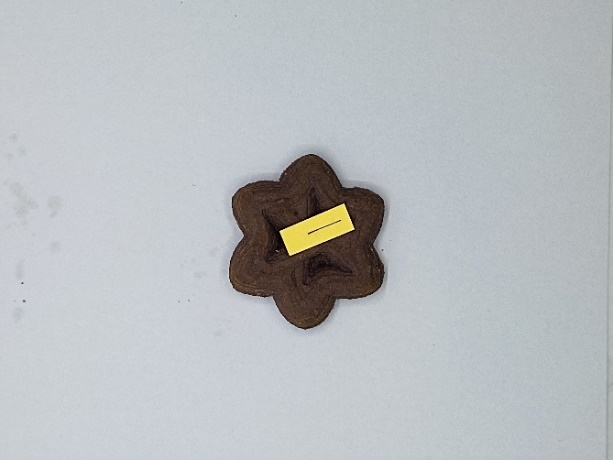 | 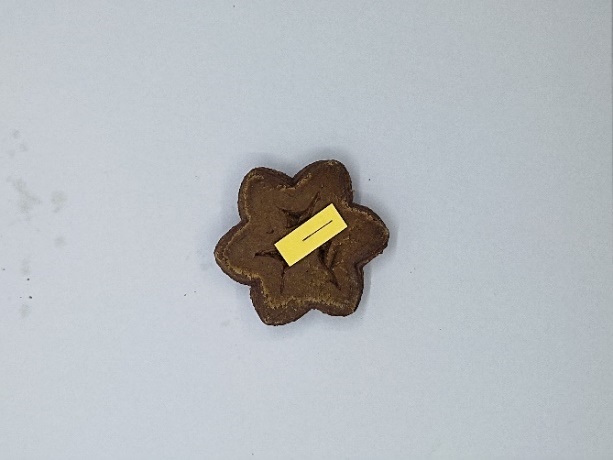 | 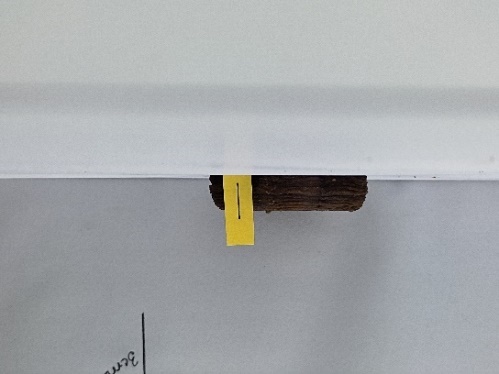 |
| 2 | 2:180:6:60 | 175.80 | 53.35 | 8.49 | 17.96 | 86.13 | 68.49 | 16.25 | 10.25 | HOE | 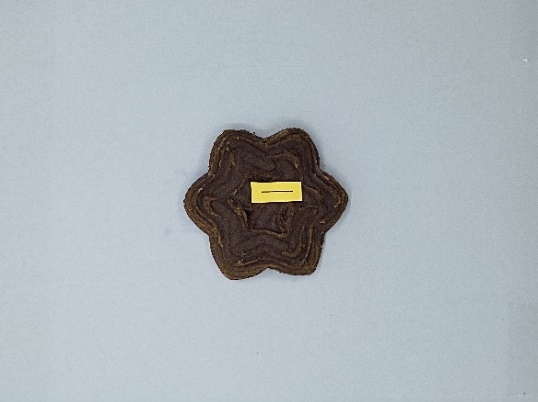 | 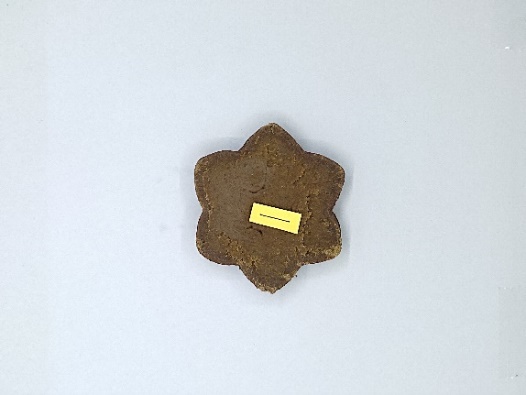 | 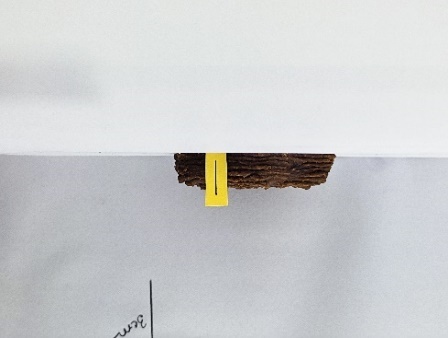 |
| 3 | 2.4:150:8:45 | 126.00 | 56.45 | 14.81 | 18.82 | 79.69 | 65.88 | 18.57 | 8.53 | HOE | 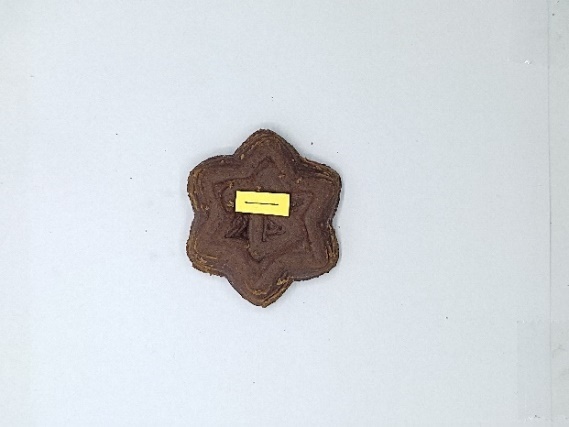 | 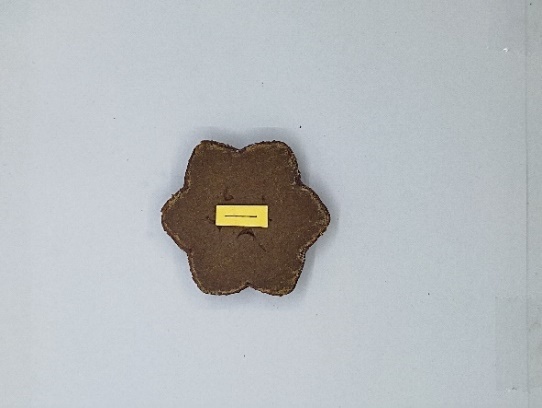 | 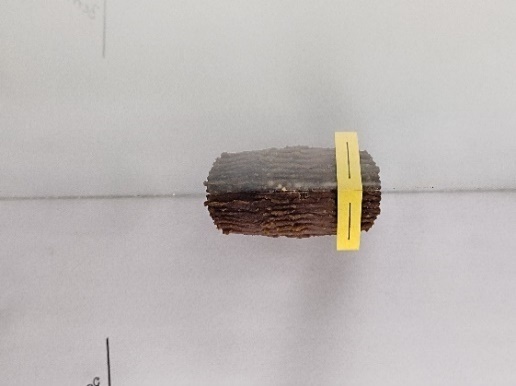 |
| 4 | 2.4:90:8:45 | 76.63 | 96.66 | 10.94 | 5.48 | 30.36 | 41.42 | 11.89 | 8.68 | IE | 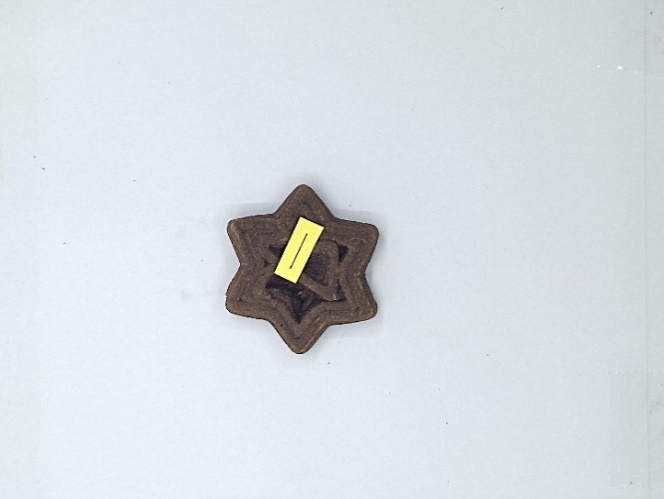 | 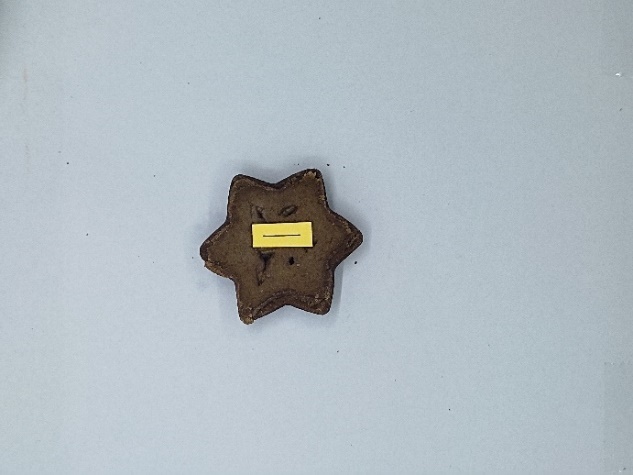 | 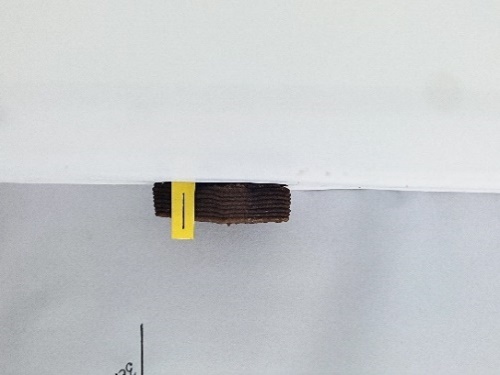 |
| 5 | 2.4:150:4:75 | 164.67 | 64.27 | 12.56 | 12.34 | 78.59 | 53.80 | 19.68 | 11.20 | HOE | 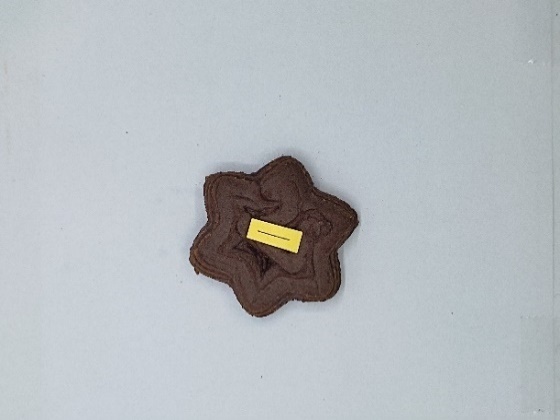 | 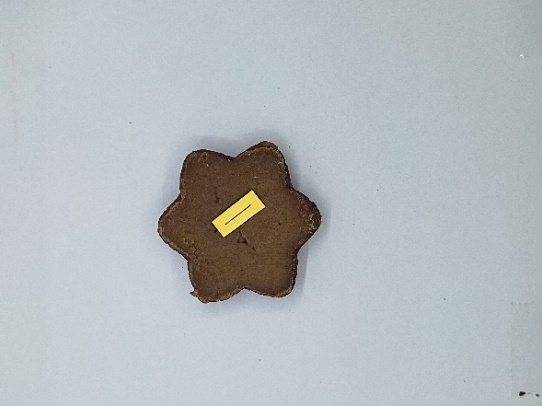 | 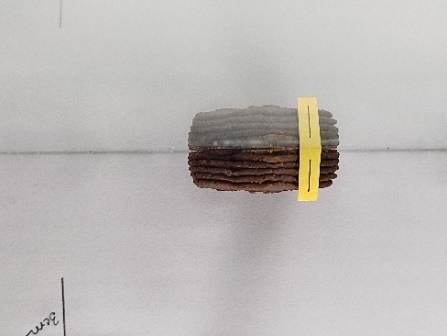 |
| 6 | 2:120:10:60 | 98.15 | 97.97 | 5.26 | 7.15 | 14.78 | 21.30 | 10.78 | 7.20 | IE | 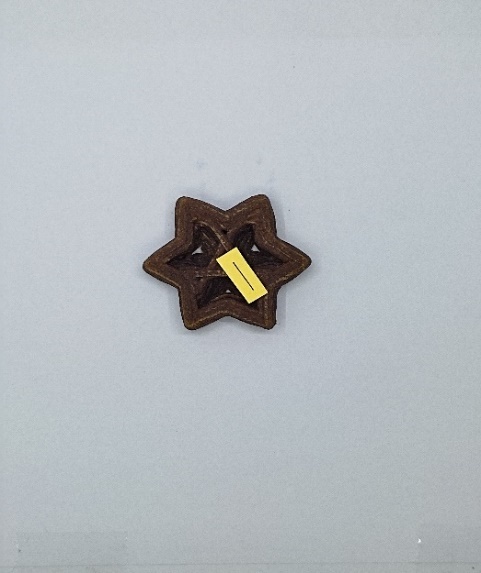 | 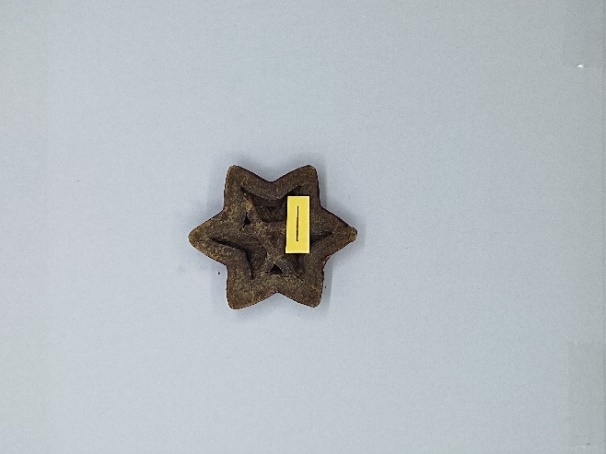 | 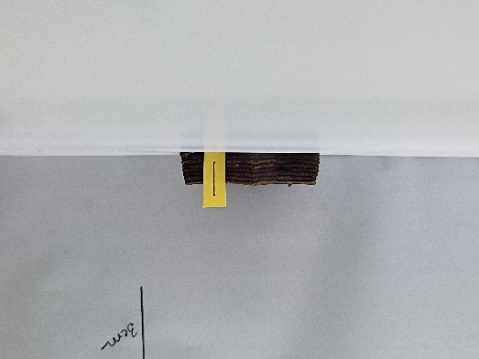 |
| 7 | 2:120:6:60 | 127.20 | 97.80 | 2.72 | 2.66 | 16.89 | 27.39 | 10.82 | 11.22 | IE | 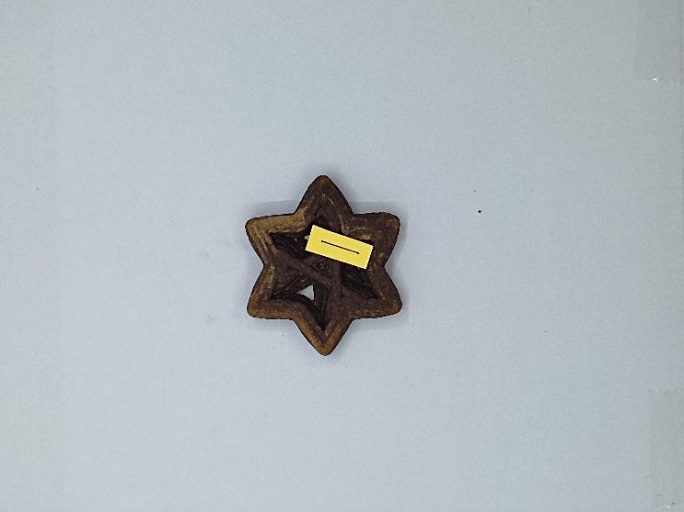 | 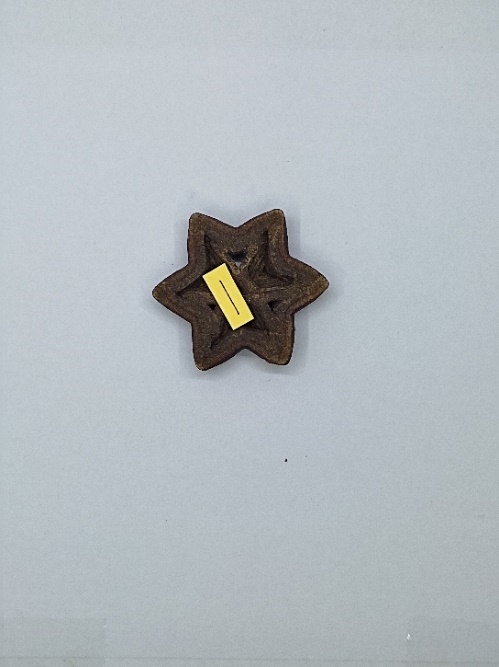 | 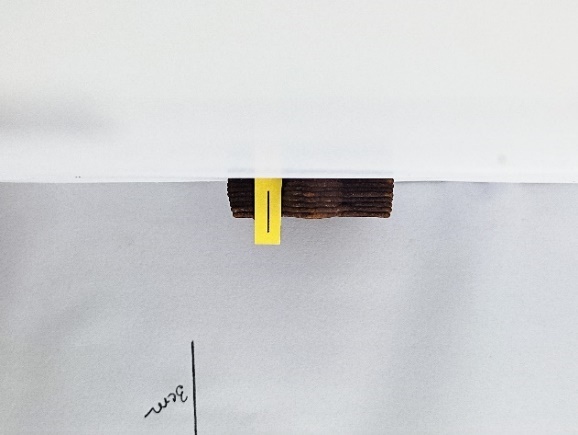 |
| 8 | 1.6:150:4:75 | 95.56 | 85.93 | 8.01 | 10.56 | 34.13 | 28.34 | 11.36 | 16.43 | MOE | 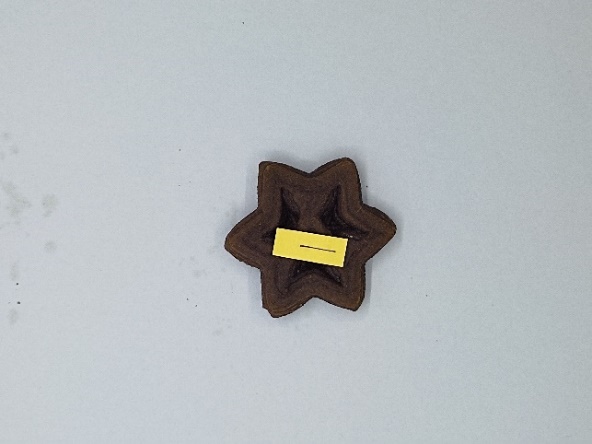 | 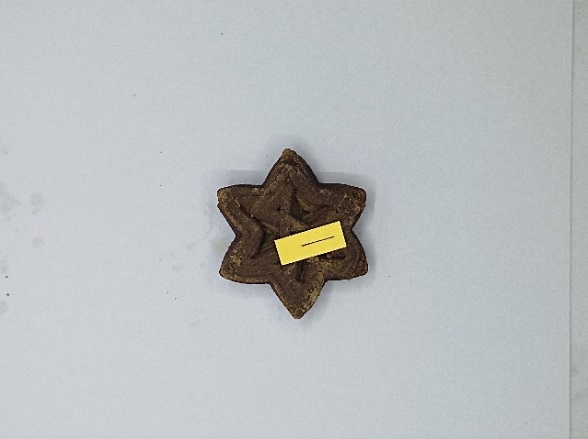 | 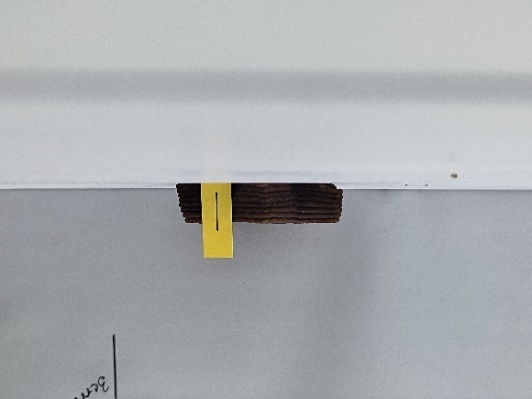 |
| 9 | 2:120:6:90 | 109.45 | 90.20 | 8.43 | 2.12 | 31.89 | 26.78 | 11.75 | 8.47 | MOE | 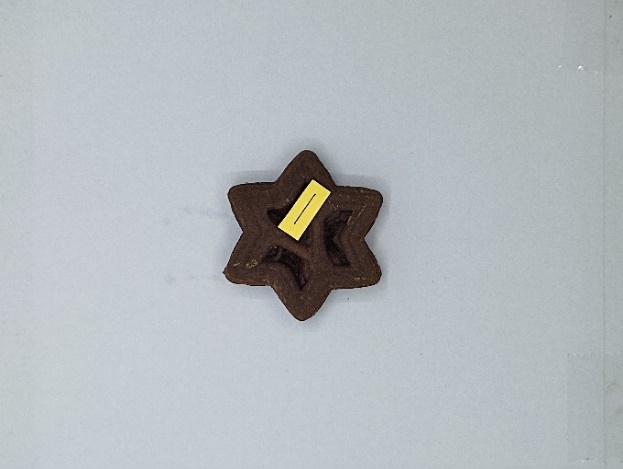 | 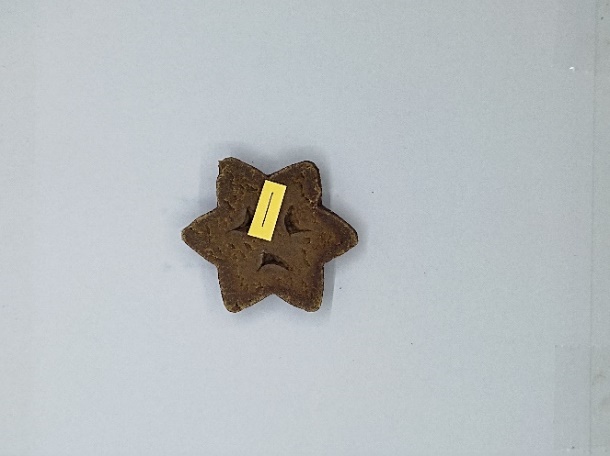 | 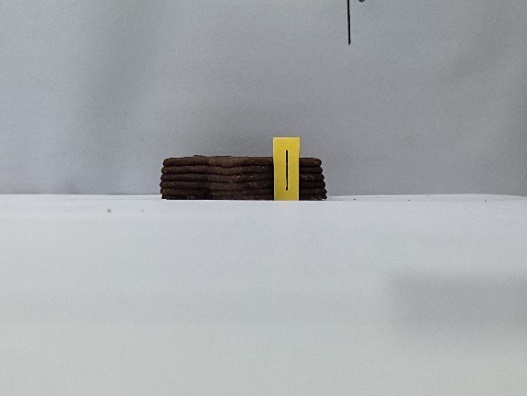 |
| 10 | 2:120:6:60 | 127.40 | 99.68 | 3.05 | 2.25 | 18.09 | 25.06 | 11.84 | 11.22 | IE | 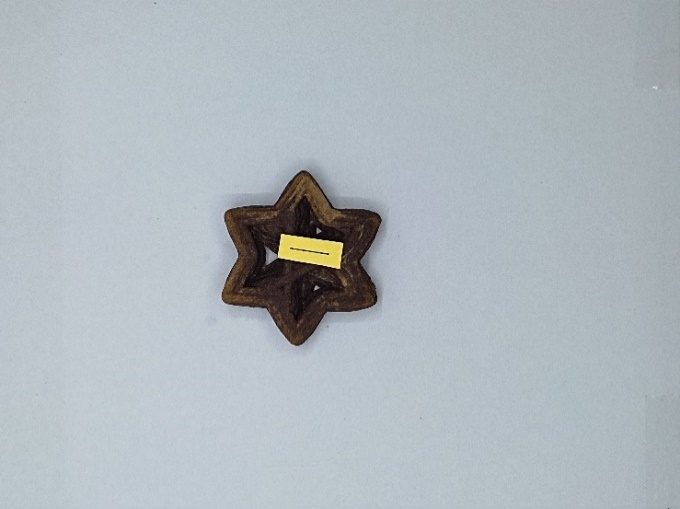 | 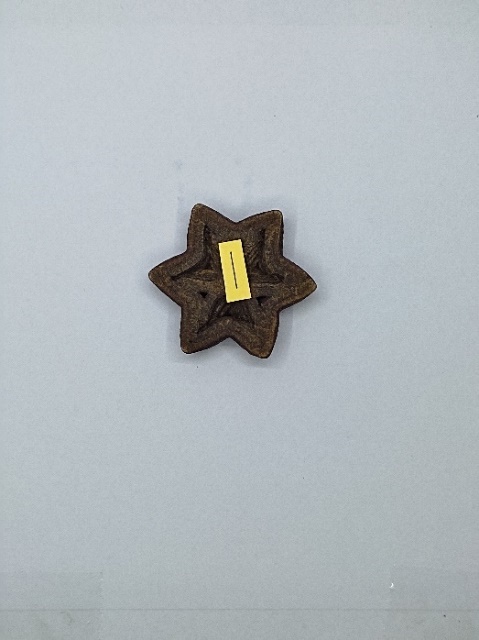 | 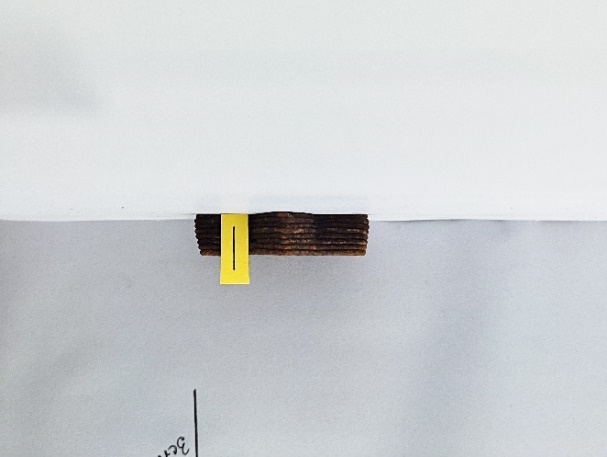 |
| 11 | 2.4:90:4:75 | 101.33 | 98.11 | 11.50 | 0.18 | 28.05 | 27.18 | 10.22 | 11.27 | IE | 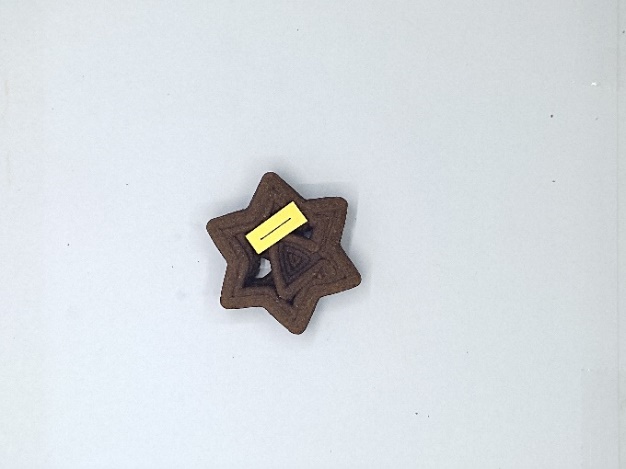 | 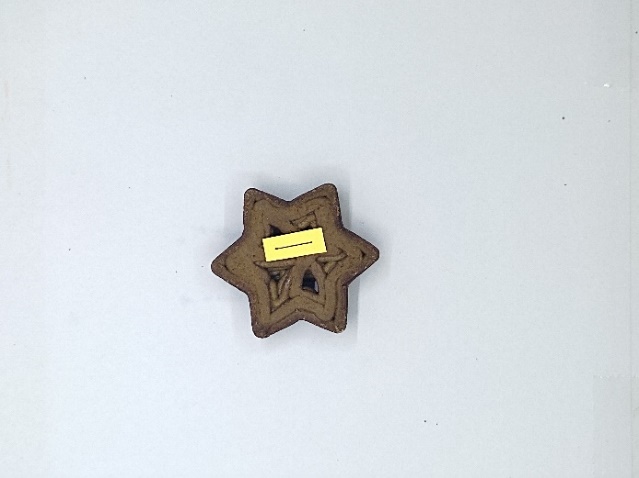 | 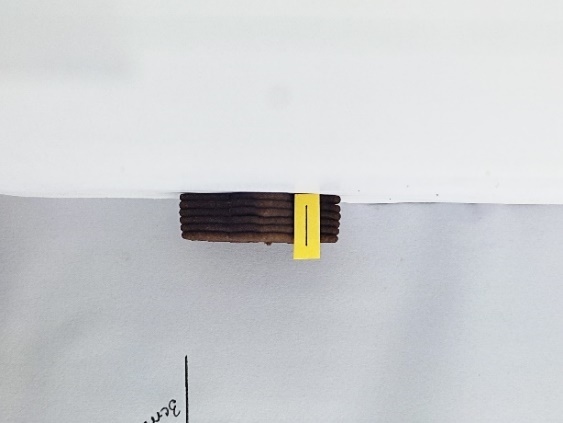 |
| 12 | 2.4:90:4:45 | 91.13 | 94.52 | 5.88 | 5.39 | 28.23 | 35.92 | 10.19 | 16.28 | IE | 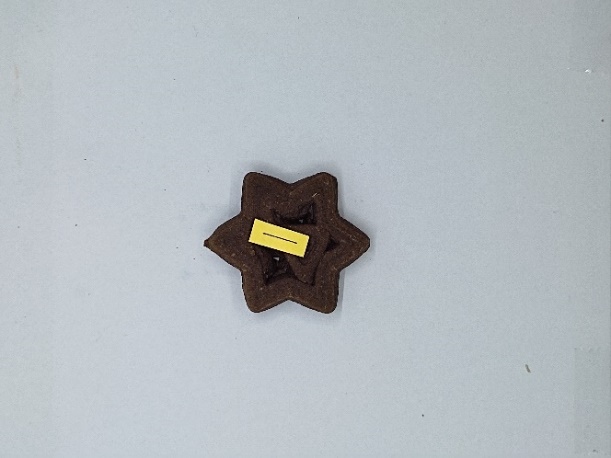 | 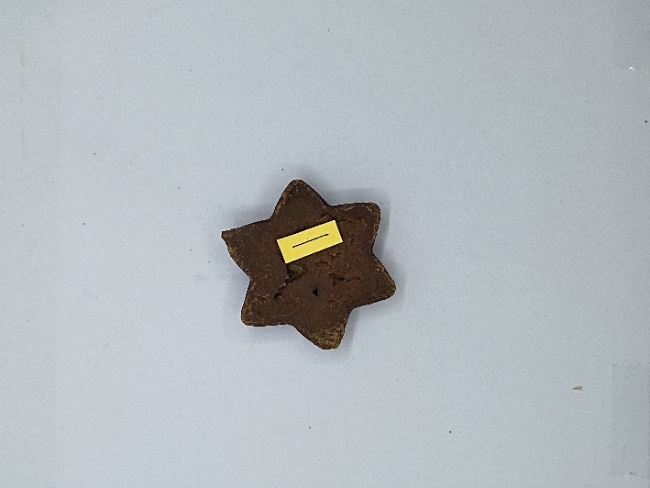 | 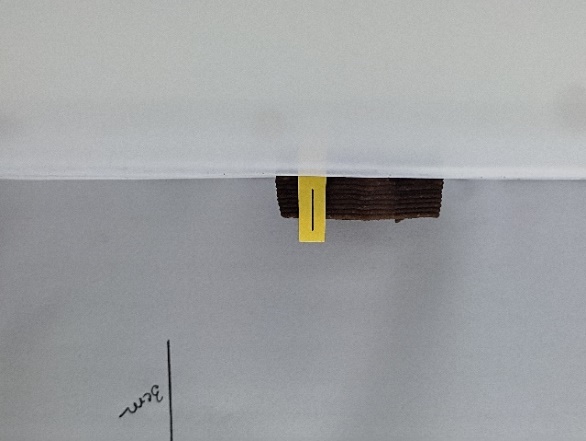 |
| 13 | 1.6:150:8:45 | 98.69 | 82.22 | 4.71 | 4.05 | 47.83 | 43.24 | 13.00 | 14.53 | MOE | 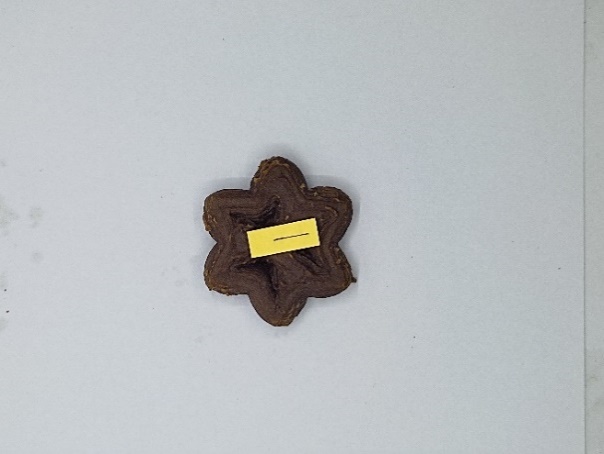 | 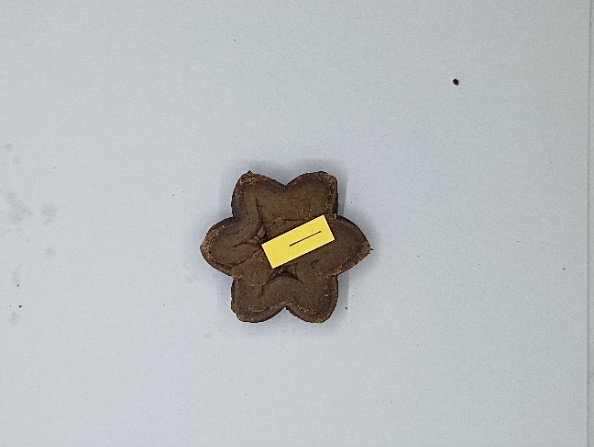 | 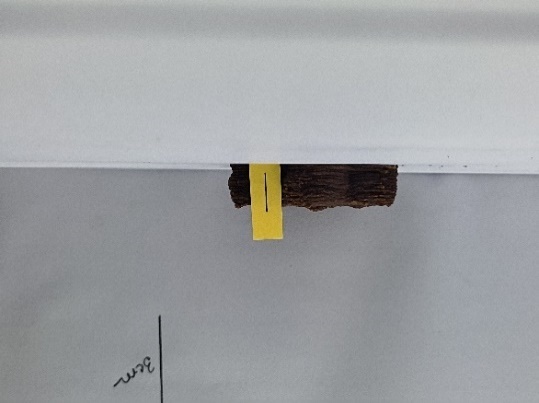 |
| 14 | 2.4:150:8:75 | 147.33 | 71.66 | 12.79 | 10.84 | 65.16 | 44.75 | 17.90 | 5.82 | HOE | 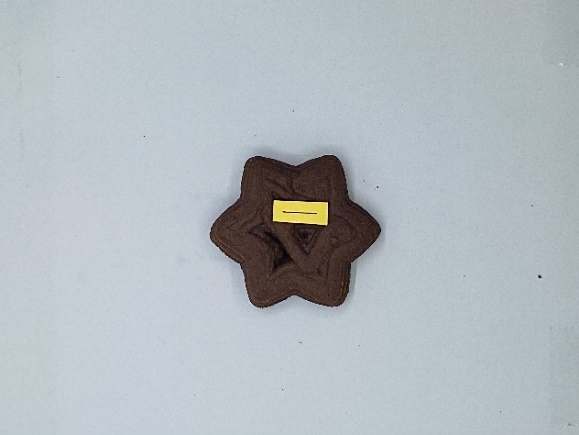 | 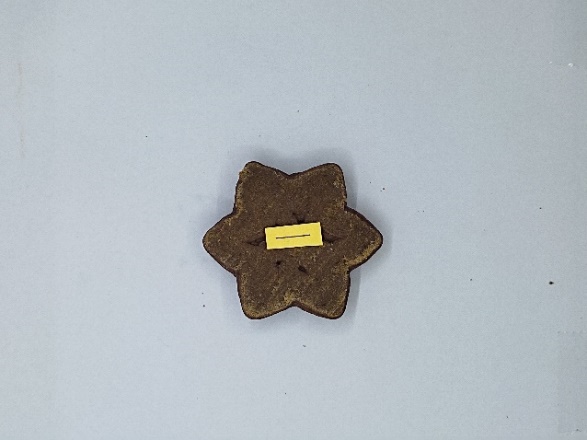 | 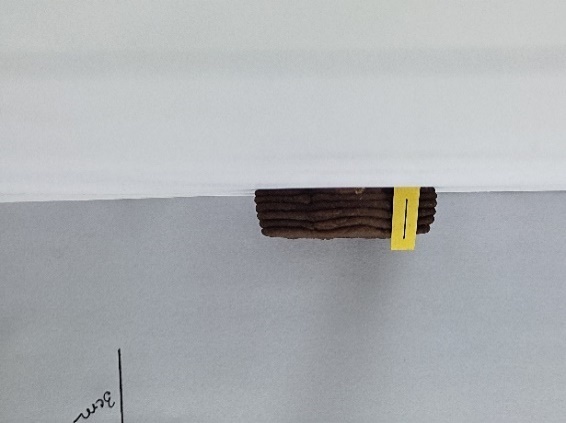 |
| 15 | 2.4:150:4:45 | 178.04 | 51.64 | 10.25 | 23.22 | 81.27 | 68.65 | 18.90 | 16.28 | HOE | 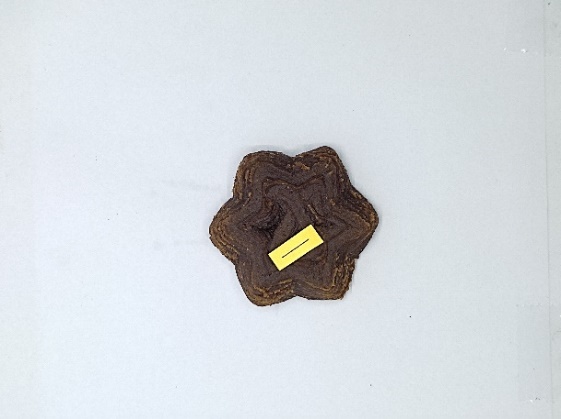 | 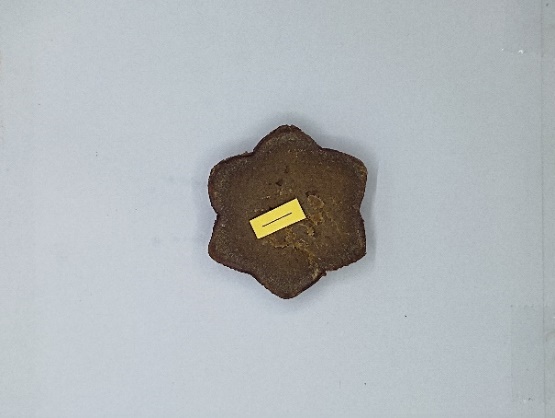 | 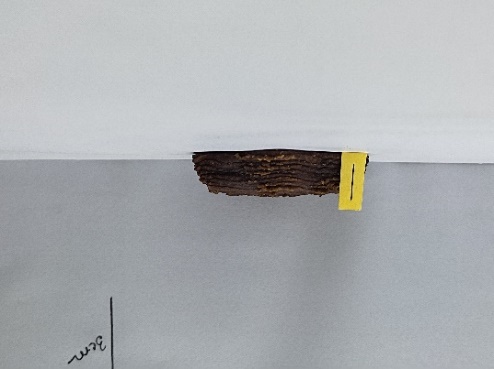 |
| 16 | 2:120:6:30 | 90.65 | 86.47 | 3.35 | 3.45 | 45.00 | 49.29 | 12.81 | 21.67 | MOE | 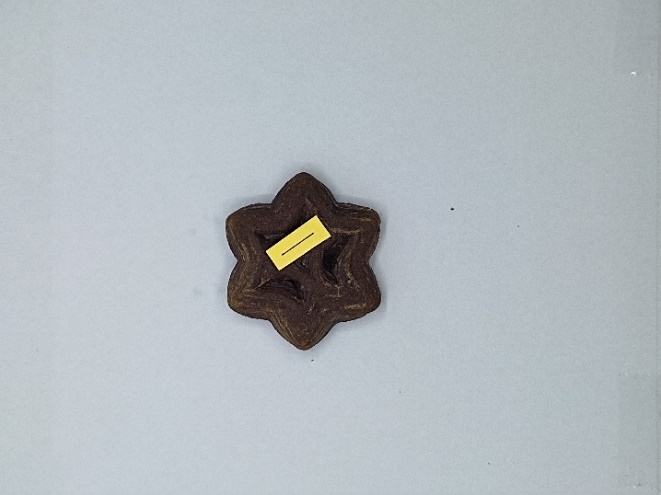 | 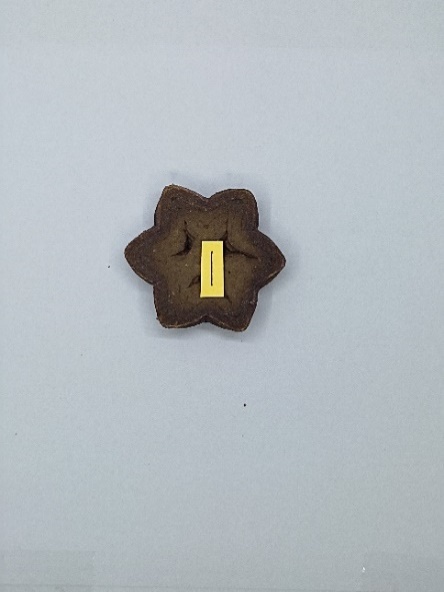 | 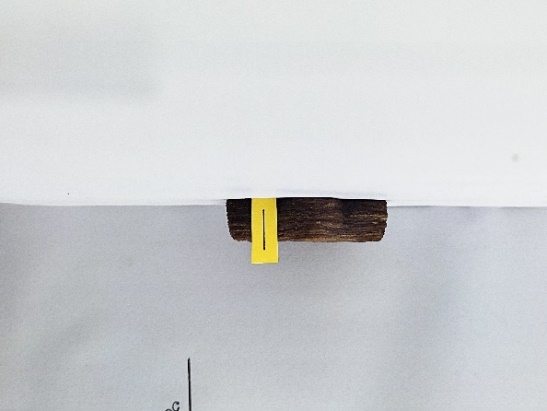 |
| 17 | 2:120:6:60 | 123.35 | 94.93 | 3.04 | 2.79 | 17.09 | 26.15 | 11.63 | 10.78 | MOE | 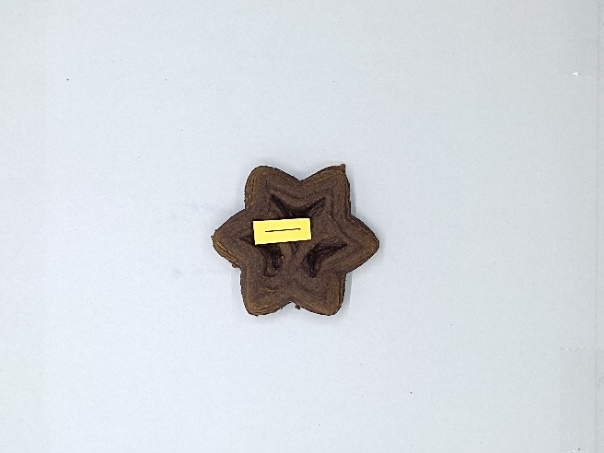 | 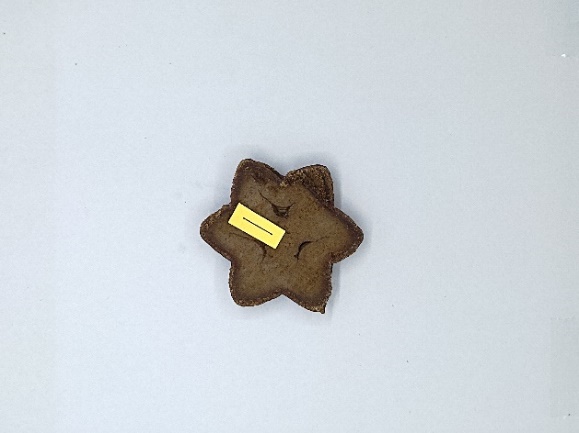 | 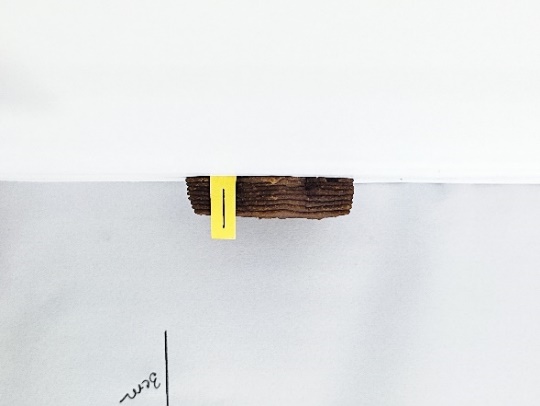 |
| 18 | 1.6:90:4:75 | 97.44 | 95.29 | 4.76 | 4.87 | 14.40 | 9.06 | 7.98 | 16.58 | IE | 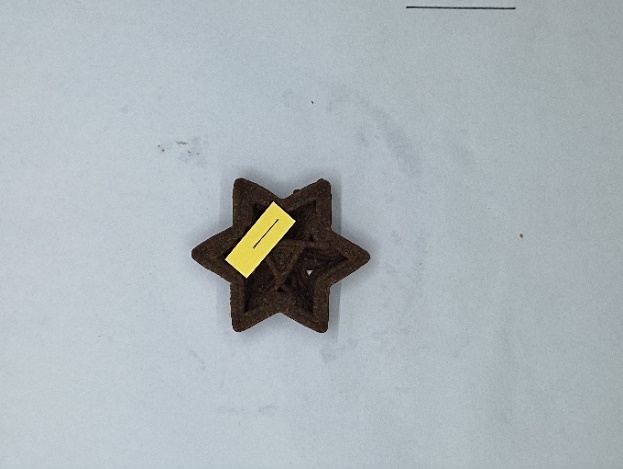 | 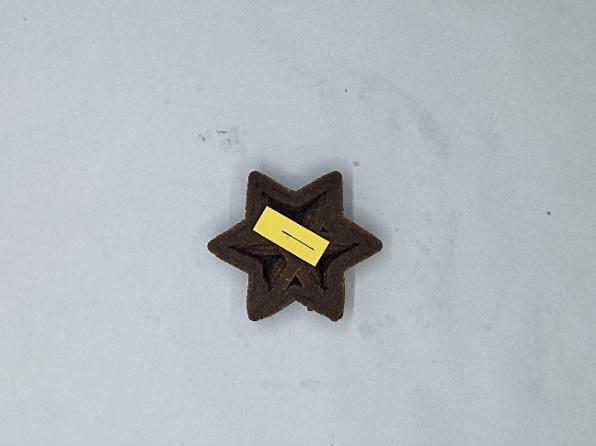 | 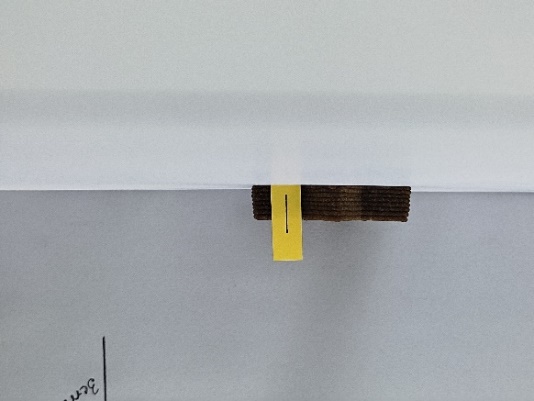 |
| 19 | 2:120:6:60 | 124.90 | 93.25 | 2.84 | 2.23 | 18.51 | 25.76 | 11.66 | 10.82 | MOE | 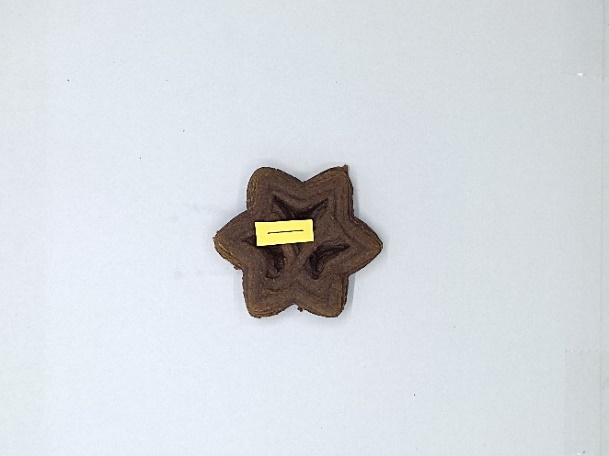 | 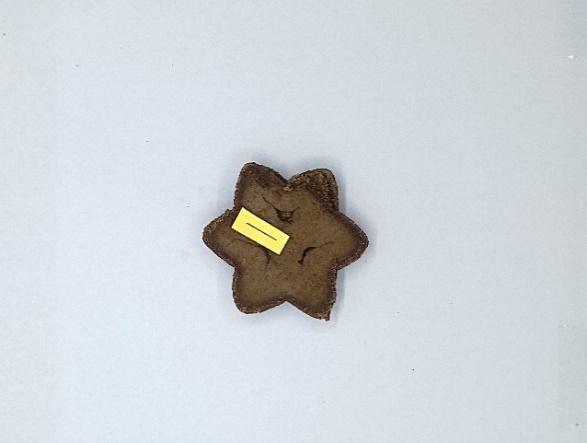 | 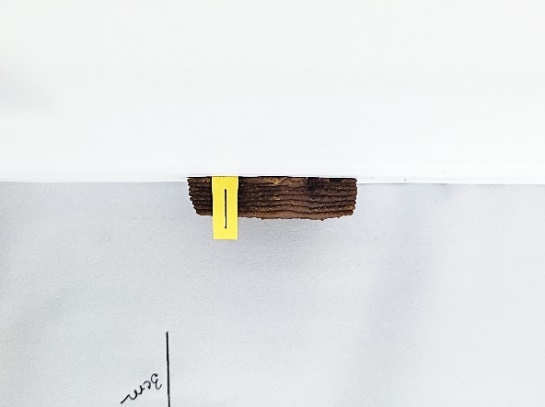 |
| 20 | 1.6:90:8:75 | 98.25 | 96.02 | 3.60 | 9.56 | 12.54 | 10.87 | 8.02 | 8.78 | IE | 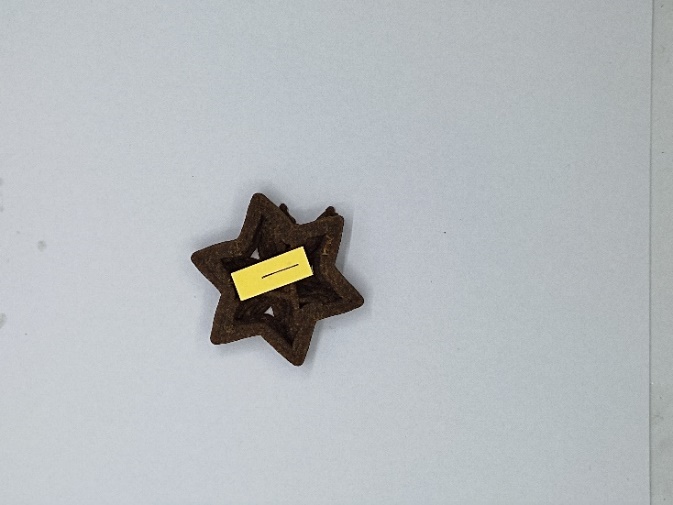 | 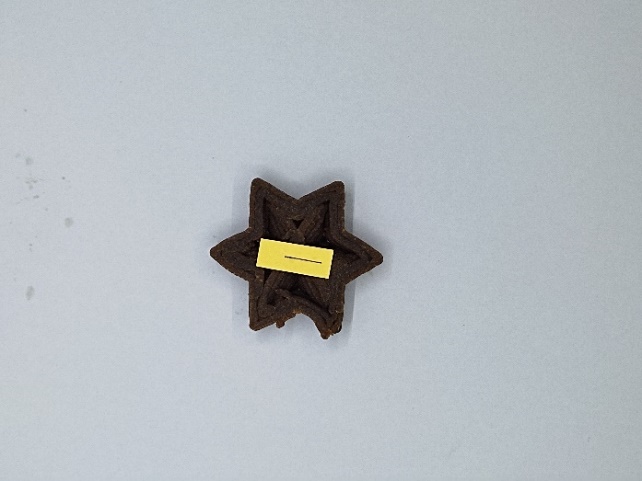 | 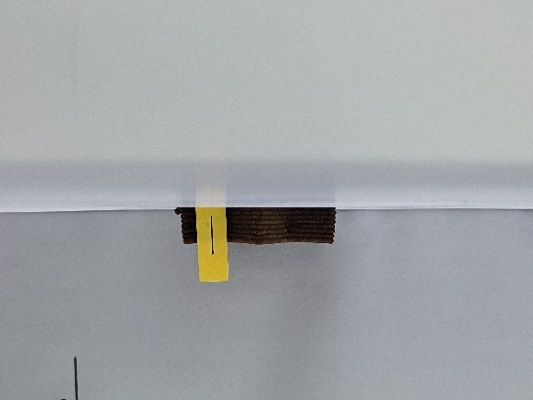 |
| 21 | 1.6:90:4:45 | 93.38 | 95.08 | 1.12 | 2.24 | 14.34 | 15.56 | 8.91 | 27.68 | IE | 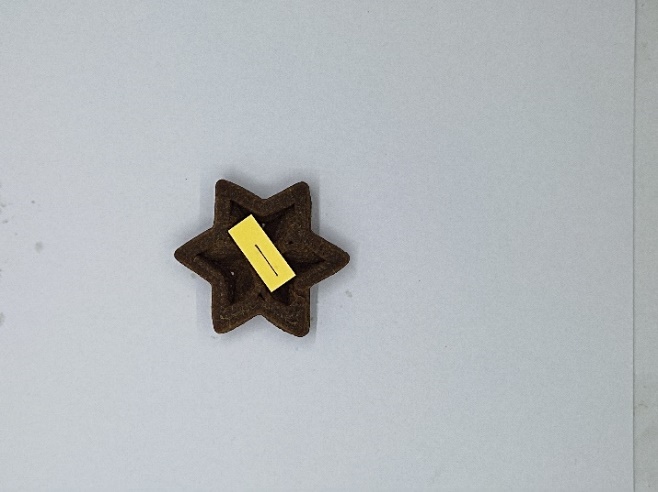 | 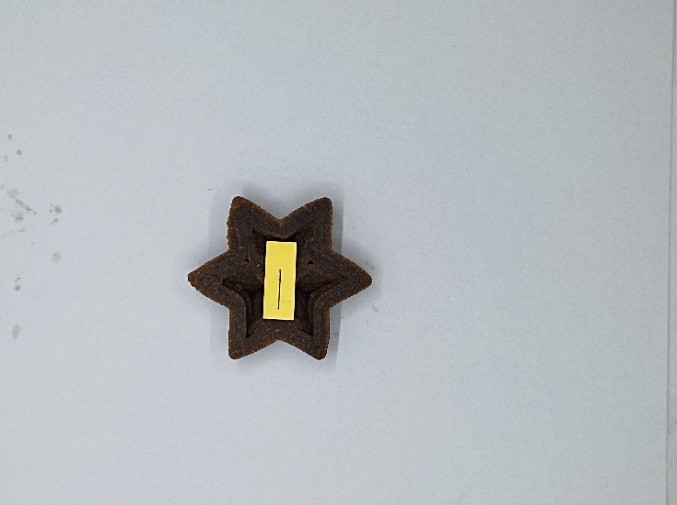 | 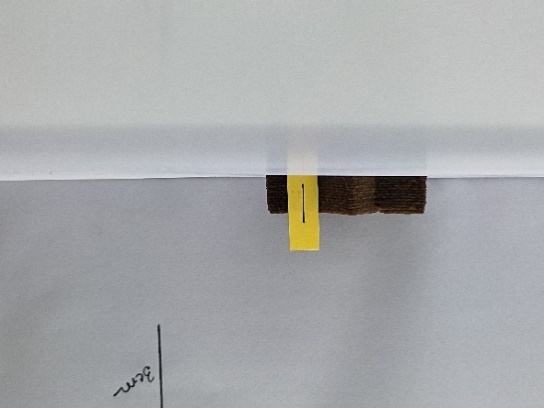 |
| 22 | 2.4:90:8:75 | 113.88 | 96.90 | 10.37 | 5.87 | 25.53 | 25.91 | 10.47 | 6.07 | IE | 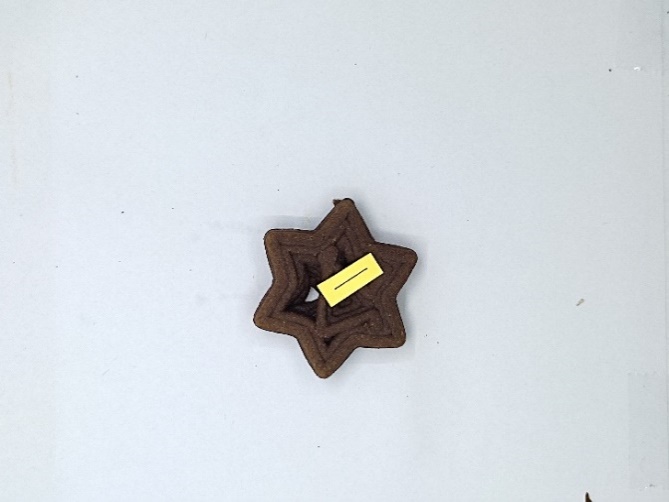 | 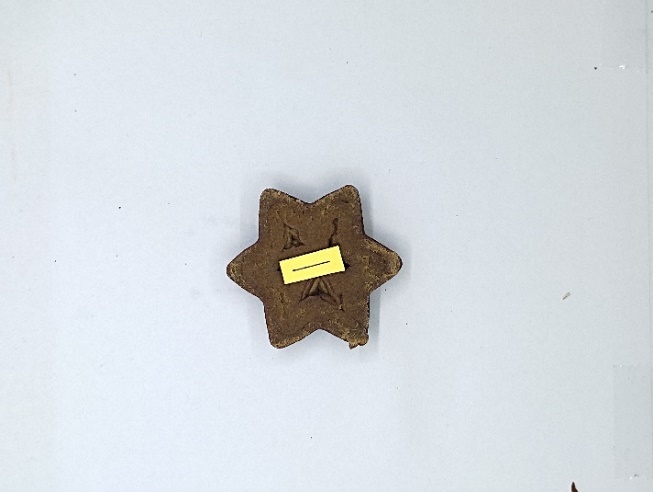 | 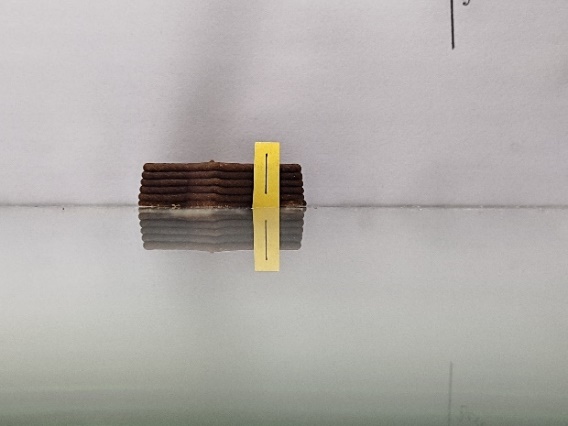 |
| 23 | 2:120:6:60 | 128.30 | 95.43 | 2.91 | 2.61 | 18.90 | 28.43 | 11.98 | 10.90 | MOE | 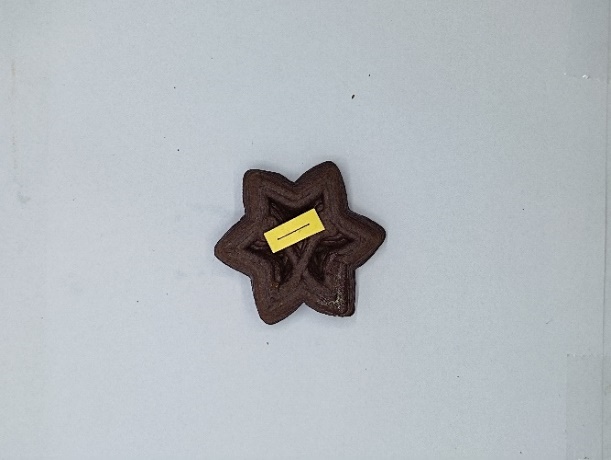 | 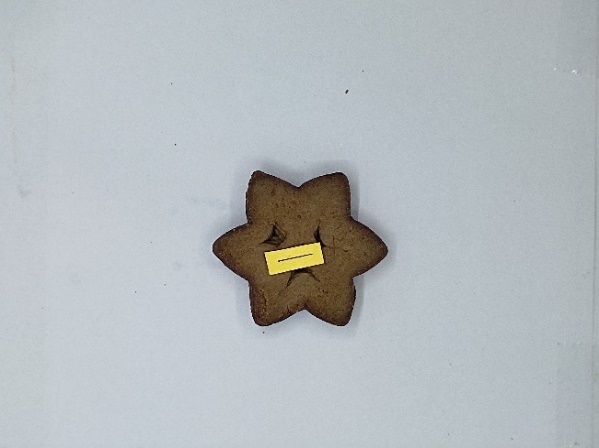 | 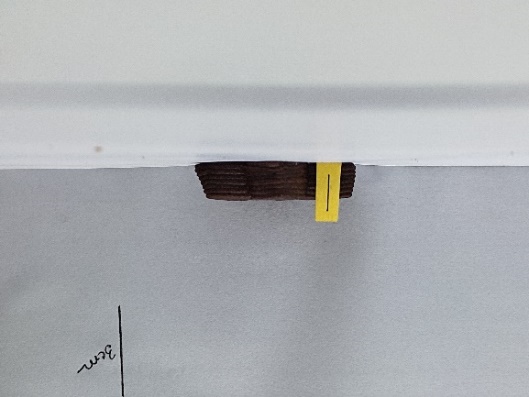 |
| 24 | 1.2:120:6:60 | 77.50 | 95.65 | 2.84 | 7.57 | 18.45 | 15.16 | 10.20 | 19.03 | IE | 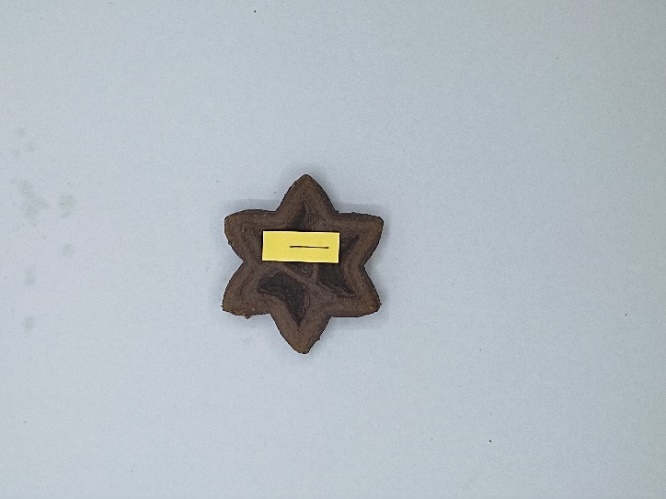 | 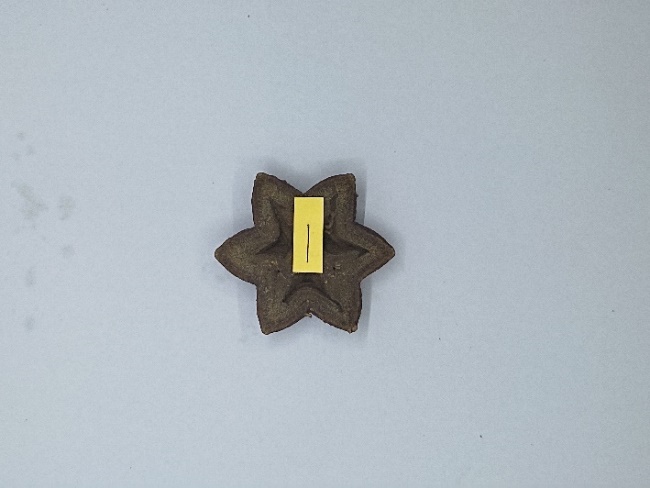 | 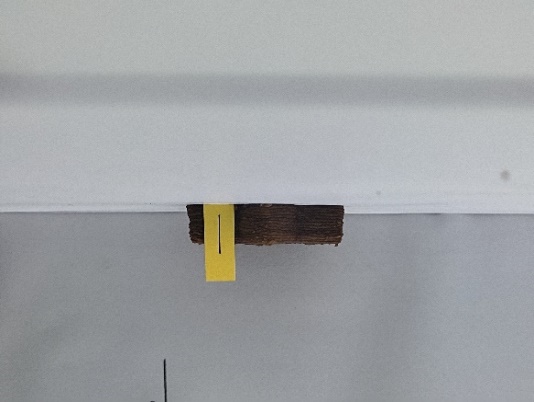 |
| 25 | 1.6:90:8:45 | 74.00 | 96.37 | 0.36 | 3.73 | 25.97 | 33.20 | 10.57 | 14.67 | IE | 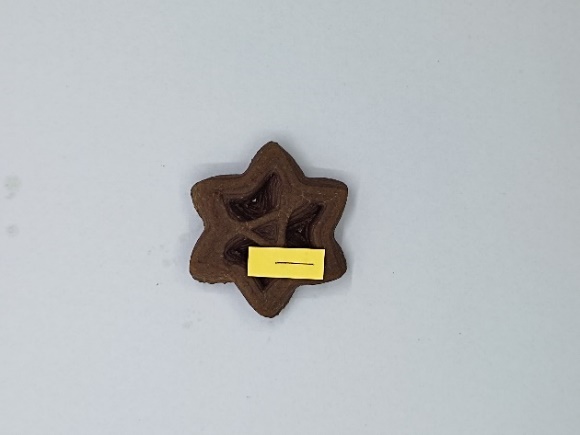 | 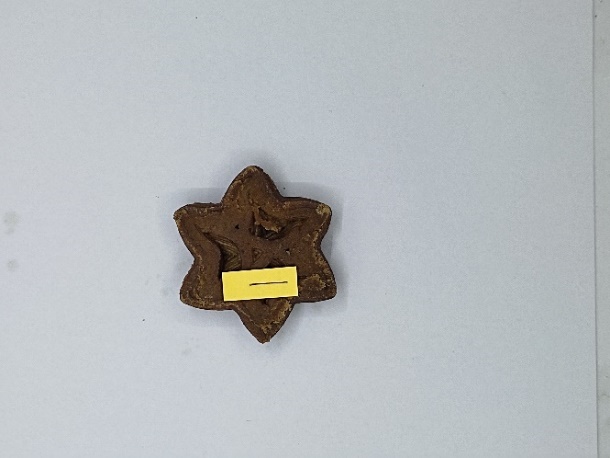 | 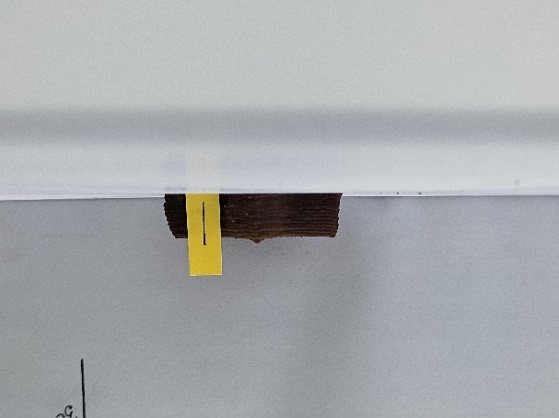 |
| 26 | 1.6:150:8:75 | 98.06 | 88.91 | 5.93 | 8.37 | 38.08 | 24.46 | 11.43 | 8.50 | MOE | 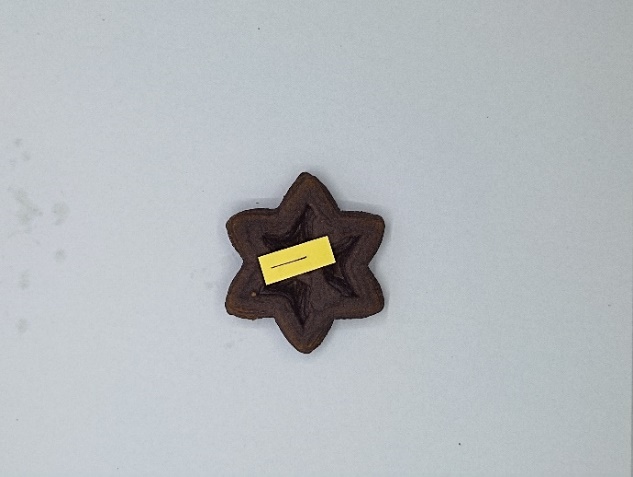 | 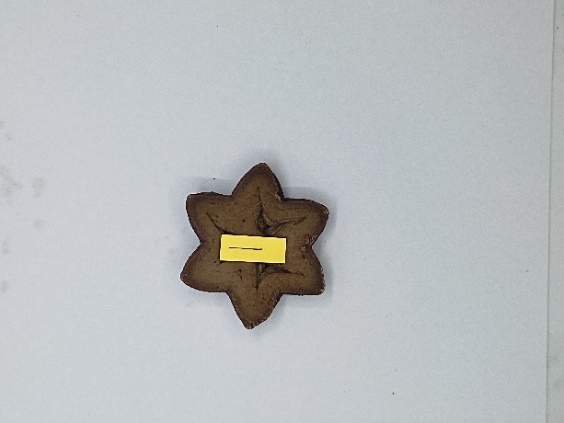 | 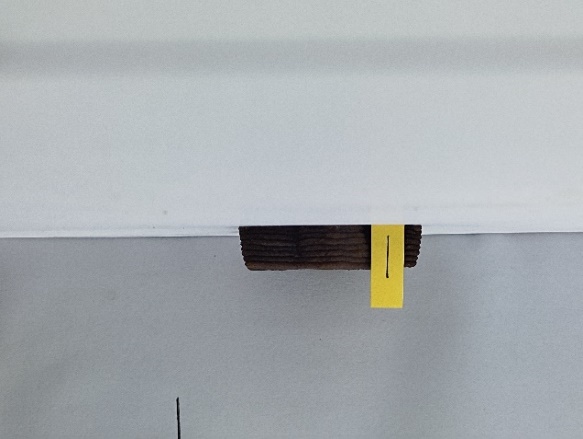 |
| 27 | 2:120:2:60 | 121.25 | 91.62 | 4.32 | 9.89 | 30.25 | 29.85 | 11.14 | 30.75 | MOE | 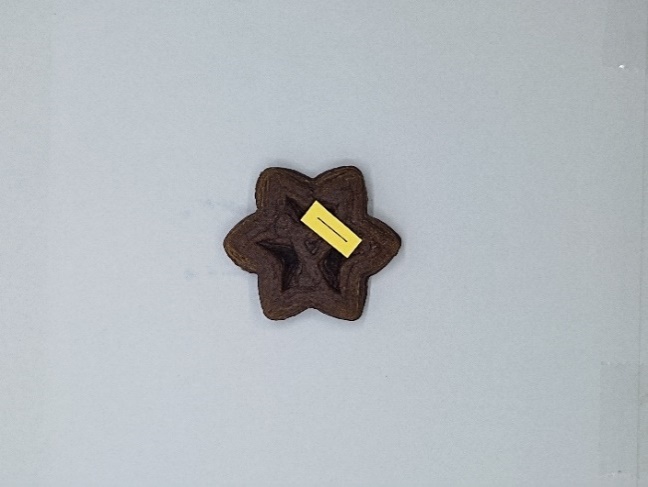 | 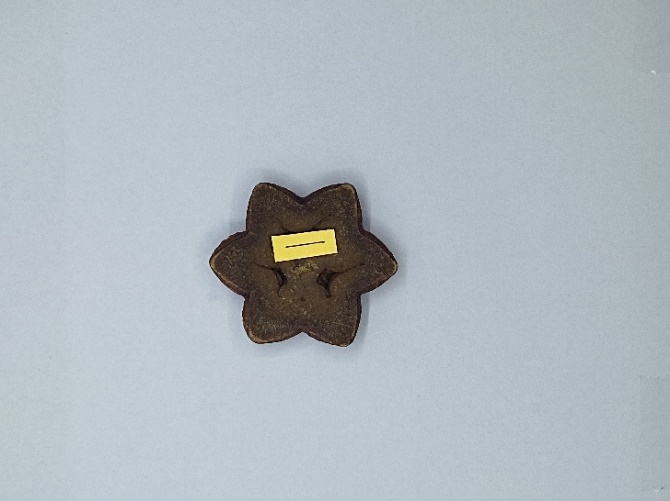 | 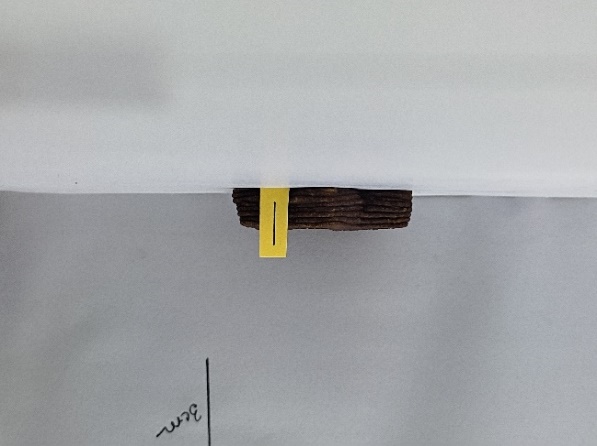 |
| 28 | 2.8:120:6:6 | 131.14 | 77.57 | 18.56 | 14.31 | 79.67 | 57.94 | 17.34 | 7.52 | HOE | 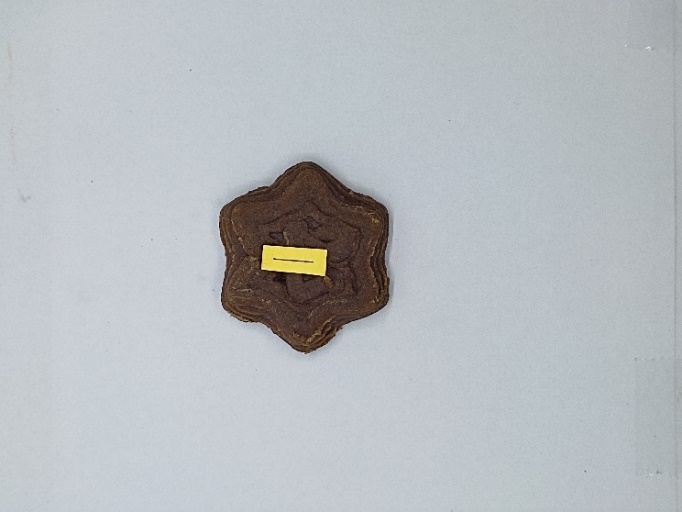 | 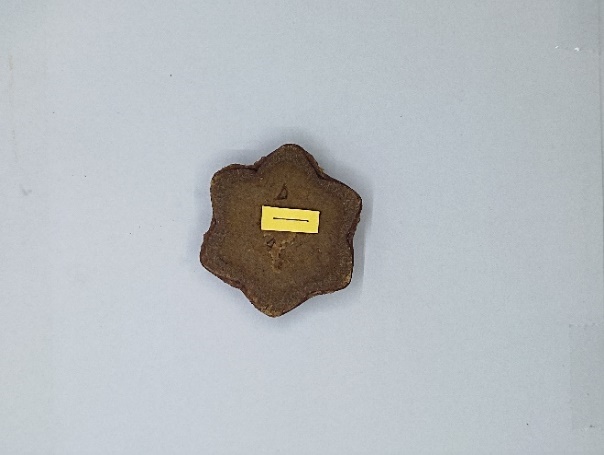 | 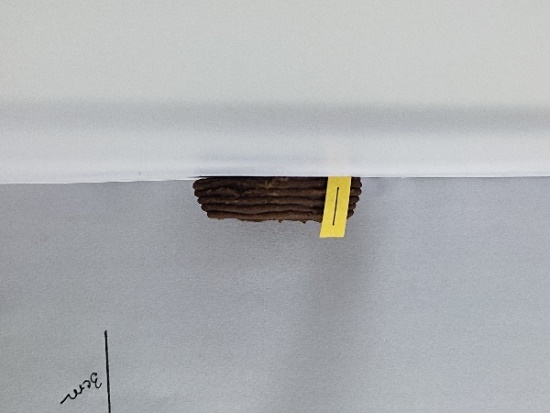 |
| 29 | 2:60:6:6 | 84.10 | 99.81 | 3.43 | 2.46 | 14.12 | 30.75 | 4.26 | 11.37 | IE | 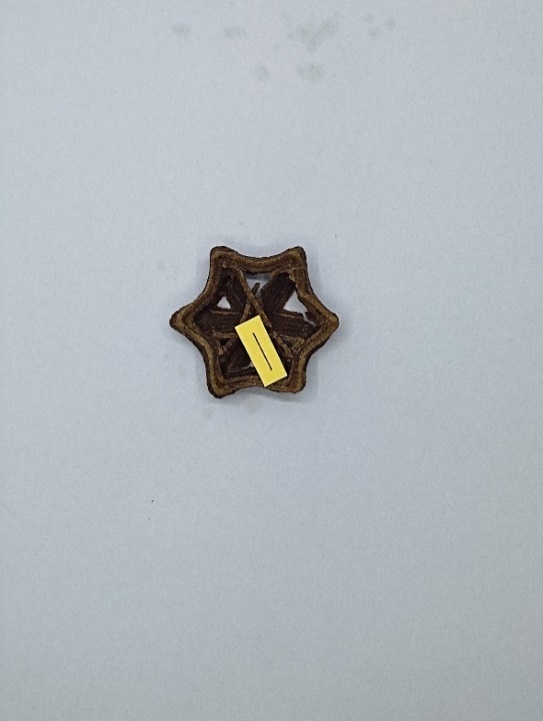 | 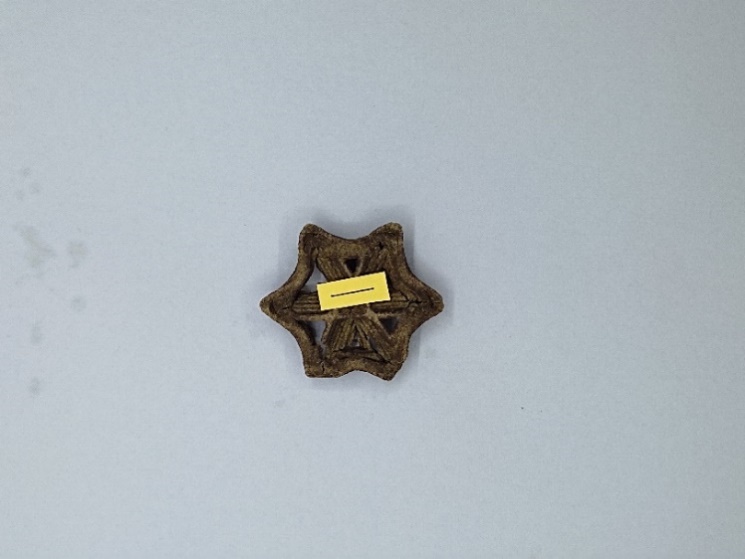 | 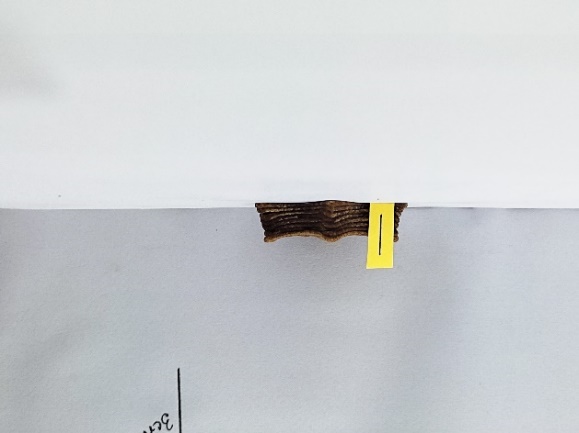 |
| 30 | 2:120:6:60 | 125.55 | 99.84 | 3.08 | 2.39 | 17.29 | 30.45 | 11.71 | 10.88 | MOE | 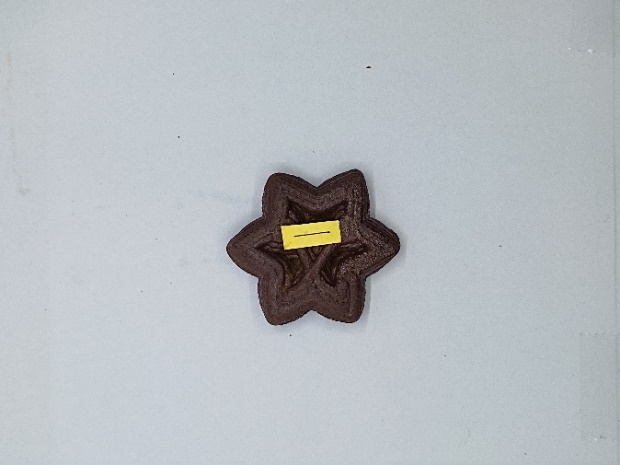 | 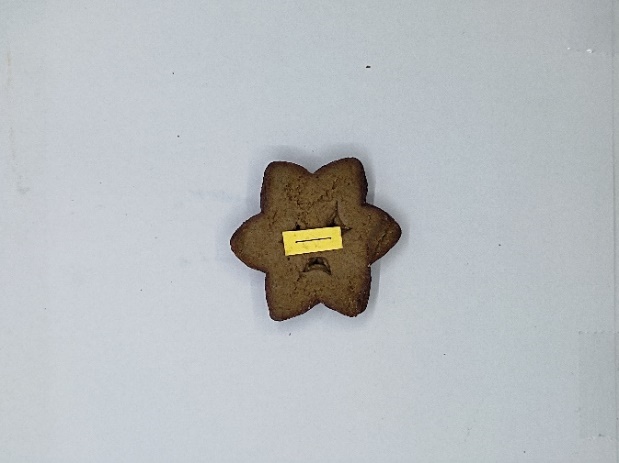 | 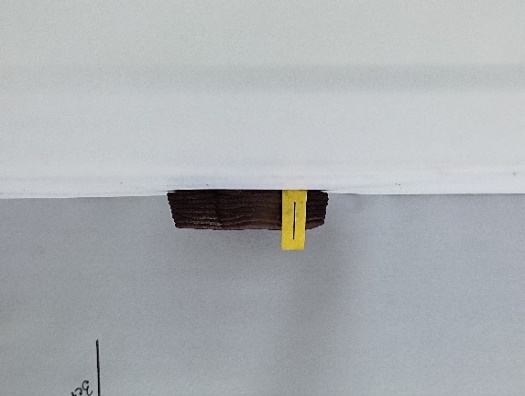 |
